# Clinical outcomes stratified by baseline functional class after initial combination therapy for pulmonary arterial hypertension

**DOI:** 10.1186/s12931-019-1180-1

**Published:** 2019-09-12

**Authors:** R. James White, Anton Vonk-Noordegraaf, Stephan Rosenkranz, Ronald J. Oudiz, Vallerie V. McLaughlin, Marius M. Hoeper, Ekkehard Grünig, Hossein-Ardeschir Ghofrani, Murali M. Chakinala, Joan A. Barberà, Christiana Blair, Jonathan Langley, Adaani E. Frost

**Affiliations:** 1 Division of Pulmonary & Critical Care Medicine, University of Rochester Medical Center, 400 Red Creek Dr, Rochester, NY 14623 USA; 2 Department of Pulmonary Medicine and Amsterdam Cardiovascular Sciences, Amsterdam UMC, Vrije Universiteit Amsterdam, Amsterdam, Netherlands; 3 Department of Cardiology and Cologne Cardiovascular Research Center (CCRC), Heart Center at the University of Cologne, Cologne, Germany; 4 Division of Cardiology, LA Biomedical Research Institute at Harbor-UCLA Medical Center, Torrance, CA USA; 5 Division of Cardiovascular Medicine, University of Michigan, Ann Arbor, MI USA; 6 Department of Respiratory Medicine, Hannover Medical School, German Center for Lung Research (DZL), Hanover, Germany; 7 Centre for pulmonary hypertension, German Center for Lung Research (DZL), Thoraxklinik at Heidelberg University Hospital, Heidelberg, Germany; 8 German Center for Lung Research (DZL), Kerckhoff Clinic, Department of Pulmonology, Universities of Giessen and Marburg Lung Center (UGMLC), Bad Nauheim, Germany; 9 Department of Medicine, Imperial College London, London, UK; 10 Division of Pulmonary & Critical Care Medicine, Washington University, St. Louis, MO USA; 11 Department of Pulmonary Medicine, Hospital Clínic-IDIBAPS, University of Barcelona, Barcelona, Spain; 12 Biomedical Research Networking Center on Respiratory Diseases (CIBERES), Madrid, Spain; 13 Gilead Sciences, Foster City, CA USA; 14 Glaxo Smith Kline, London, UK; 15 Houston Methodist Hospital, Institute for Academic Medicine, Houston, TX USA

**Keywords:** Pulmonary hypertension, Research-clinical, Combination therapy

## Abstract

**Background:**

Initial combination therapy with ambrisentan and tadalafil reduced the risk of clinical failure events for treatment-naïve participants with pulmonary arterial hypertension (PAH) as compared to monotherapy. Previous studies in PAH have demonstrated greater treatment benefits in more symptomatic participants.

**Methods:**

AMBITION was an event-driven, double-blind study in which participants were randomized 2:1:1 to once-daily initial combination therapy with ambrisentan 10 mg plus tadalafil 40 mg, ambrisentan 10 mg plus placebo, or tadalafil 40 mg plus placebo. In this pre-specified subgroup analysis, we compared the efficacy data between those with functional class (FC) II vs. FC III symptoms at baseline.

**Results:**

This analysis included 500 participants in the previously defined primary analysis set (*n* = 155 FC II, *n* = 345 FC III). Comparing combination therapy to pooled monotherapy, the risk of clinical failure events was reduced by 79% (hazard ratio, 0.21 [95% confidence interval: 0.071, 0.63]) for FC II patients and 42% (hazard ratio, 0.58 [95% confidence interval: 0.39, 0.86]) for FC III patients. In a post-hoc analysis, the risk of first hospitalization for worsening PAH was also reduced by combination therapy, particularly for FC II patients (0 combination vs. 11 [14%] pooled monotherapy). Adverse events were frequent but comparable between the subgroups.

**Conclusions:**

Treatment benefit from initial combination therapy appeared at least as great for FC II as for FC III participants. Hospitalizations for worsening PAH were not observed in FC II participants assigned to combination. The present data support an initial combination strategy for newly diagnosed patients even when symptoms are less severe.

Funded by Gilead Sciences, Inc. and GlaxoSmithKline; AMBITION ClinicalTrials.gov number, NCT01178073.

**Supplementary information:**

**Supplementary information** accompanies this paper at 10.1186/s12931-019-1180-1.

## Background

Pulmonary arterial hypertension (PAH) is a progressive disease of the pulmonary vasculature characterized by vasoconstriction, vascular inflammation and fibrosis, and in situ thrombosis [1]. The etiology is poorly understood, and many patients progress to right ventricular failure and death despite current therapy [2]. Parenteral prostacyclin analogues are uniformly recommended to treat patients with high-risk manifestations, but the optimal use of oral therapies in low- or intermediate-risk patients remains an important area of research [3]. Sequential combination therapy delayed clinical worsening in the registration trials for macitentan [4], riociguat [5], and selexipag [6]. In each of these studies, participants with FC III symptoms drove the primary treatment effect.

The AMBITION study demonstrated a 50% reduction in the risk of clinical failure for treatment naïve PAH participants who began initial combination therapy (ambrisentan and tadalafil) as compared to those assigned monotherapy [7]. The benefit of initial combination therapy was primarily attributable to a reduction in PAH-related hospitalizations. The present paper describes a prespecified subgroup analysis which planned to evaluate the primary outcome in participants with FC II symptoms as compared to those with more advanced FC III symptoms. Based on previous studies, we hypothesized that the statistically and clinically relevant benefits of initial combination therapy would be largely attributable to those with FC III symptoms at baseline.

## Methods

### Participants and treatments

Details of the study have been reported previously [7]. Each enrolling site had institutional review before consenting participants, and each participant provided written informed consent before study procedures. Briefly, participants were treatment-naïve and symptomatic patients with idiopathic or heritable PAH; or PAH associated with connective tissue disease, drugs or toxins, stable HIV infection, or congenital heart defects repaired > 1 year prior to screening. Following initial enrollment of ~ 150 patients, a blinded review of demographic data revealed a greater than anticipated prevalence of risk factors for left ventricular diastolic dysfunction. Protocol amendment 2 therefore restricted enrollment to participants with no more than 2 risk factors for left ventricular diastolic dysfunction. This amendment also specified a more rigorous hemodynamic definition for participants with pulmonary capillary wedge pressures between 13 and 15 mmHg. Participants meeting amendment 2 criteria have been referred to as the primary analysis set (PAS, *n = 500*), and they are the focus of this analysis.

This event-driven study required 105 events in the PAS for ~ 97% power to detect a 53% reduction in hazard rate between combination therapy and pooled monotherapy. The primary endpoint was time to first clinical failure event (TtCF). The components of TtCF are provided in Table 1; a blinded, independent committee adjudicated all components (and all hospitalizations through the end of study.)

**Table 1:** Components and Definitions of the Primary Endpoint

Component	Definition
Death (all-cause)	Certificate of death
Hospitalization for worsening PAH	Adjudicated and defined as any hospitalization for worsening PAH, lung or heart/lung transplant, atrial septostomy; participants who initiated parenteral prostanoid therapy were included in this group
Disease progression	Adjudicated and defined as a (decrease of > 15% from baseline in 6MWD combined with WHO FC III or IV symptoms) at 2 consecutive visits separated by ≥14 days
Unsatisfactory long-term clinical response	Adjudicated and requiring participation in the study for ≥6 months; defined as sustained WHO FC III symptoms AND any decrease from baseline in 6MWD at 2 consecutive visits separated by ≥14 days

From New England Journal of Medicine. Galiè N, Barberà JA, Frost AE, Ghofrani HA, Hoeper MM, McLaughlin VV, Peacock AJ, Simonneau G, Vachiery JL, Grünig E, Oudiz RJ, Vonk-Noordegraaf A, White RJ, Blair C, Gillies H, Miller KL, Harris JH, Langley J, Rubin LJ; AMBITION Investigators. Initial use of ambrisentan plus tadalafil in pulmonary arterial hypertension. Volume 373, Pages 834–844. Copyright© (2015) Massachusetts Medical Society. Reprinted with permission from Massachusetts Medical Society

*6MWD* 6-min walk distance, *FC* Functional class, *PAH* Pulmonary arterial hypertension, *WHO* World Health Organization

Randomization was 2:1:1 to combination therapy or monotherapy (ambrisentan or tadalafil monotherapy), stratified by underlying etiology of PAH and baseline FC. The study was conducted in accordance with the Declaration of Helsinki and its amendments (ClinicalTrials.gov number, NCT01178073). The protocol was approved by the institutional review board at each enrolling center (Additional file 1).

### Statistical analysis

Analyses are presented by baseline FC and include prespecified analyses on TtCF, 6-min walk distance, and satisfactory clinical response (other analyses were post-hoc following the initial observations). The Kaplan-Meier product limit method was used to generate survival curves for TtCF and time to first hospitalization for worsening PAH; treatment groups were compared using the stratified log-rank test. Cox proportional-hazards regression models were used to calculate the hazard ratios and 95% confidence intervals. As typical for similar analyses, the alpha level for the interaction between primary outcome and subgroup was set a priori at 0.1. Detailed statistical methods and imputation strategies are presented in the Additional file 1.

## Results

### Participants

**Fig. 1:**
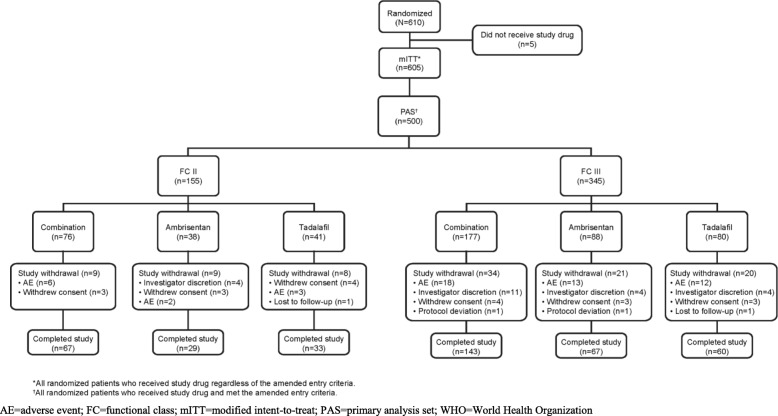
Patient Disposition by WHO Functional Class and Treatment Group

**Fig. 2:**
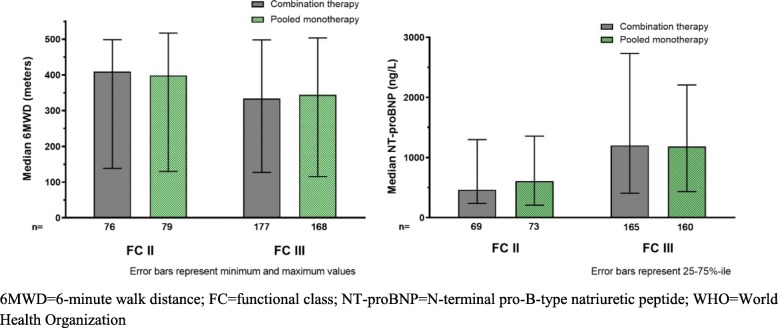
Baseline Values of 6MWD (**a**) and NT-proBNP (**b**) by WHO Functional Class and Treatment Group

Patient disposition is shown in Fig. 1. FC II participants were equally distributed in each treatment group (Table 2) by design. Participants with FC III symptoms tended to be older, and there were more participants with CTD-associated PAH in FC III as compared to FC II (in all treatment groups). More males with FC II symptoms were randomized to combination therapy; otherwise treatment assignments were balanced. Baseline 6MWD and NT-proBNP levels were similar among the FC II treatment groups and noticeably different from the FC III participants, consistent with the investigator assigned FC (Fig. 2). Importantly, despite having mild functional limitations, median 6MWD and NT-proBNP in the FC II group suggested that many participants would be at ‘intermediate risk’ for mortality according to ESC/ERS guidelines [3]. Table 2 also illustrates that pulmonary vascular resistance tended to be higher in those assessed as FC III although the differences were not as prominent as for 6MWD and NT-proBNP.

**Table 2:** Demographic and Baseline Characteristics by WHO Functional Class and Treatment Group (Primary Analysis Set)

	Combination Therapy		Pooled Monotherapy		Ambrisentan Monotherapy		Tadalafil Monotherapy		Total (*N* = 500)	
	(*n* = 253)		(*n* = 247)		(*n* = 126)		(*n* = 121)			
Baseline Functional Class	FC II	FC III	FC II	FC III	FC II	FC III	FC II	FC III	FC II	FC III
Patients (*n*)	76	177	79	168	38	88	41	80	155	345
Age, years										
Mean	50	56	53	55	52	55	54	55	52	56
SD	16	13	15	15	15	14	15	15	15	14
Female	66%	78%	80%	82%	82%	78%	78%	85%	73%	80%
North America^b^	53%	43%	53%	42%	53%	35%	54%	49%	53%	42%
Type of PAH										
Idiopathic/ Heritable PAH	53%	53%	58%	59%	58%	60%	59%	58%	55%	56%
PAH associated with connective tissue disease^a^	34%	44%	28%	37%	32%	36%	24%	38%	31%	40%
PAH associated with congenital heart disease^a^	7%	0	4%	1%	3%	0	5%	1%	5%	< 1%
Hemodynamic variables (mean ± SD)										
Right atrial pressure (mmHg)	*N* = 76 7 ± 4	*N* = 176 8 ± 5	*N* = 79 7 ± 5	*N* = 167 8 ± 5	*N* = 38 7 ± 4	*N* = 87 8 ± 5	*N* = 41 8 ± 5	*N* = 80 9 ± 5	*N* = 155 7 ± 4	*N* = 343 8 ± 5
Pulmonary artery pressure (mmHg)	*N* = 76 47 ± 14	*N* = 177 49 ± 12	*N* = 79 46 ± 12	*N* = 168 51 ± 12	*N* = 38 47 ± 13	*N* = 88 52 ± 12	N = 41 45 ± 12	*N* = 80 50 ± 13	*N* = 155 46 ± 13	*N* = 345 50 ± 12
Pulmonary capillary wedge pressure (mmHg)	*N* = 72 9 ± 3	*N* = 172 8 ± 3	*N* = 77 9 ± 3	*N* = 159 9 ± 3	*N* = 38 8 ± 3	*N* = 83 9 ± 3	*N* = 39 10 ± 3	*N* = 76 9 ± 4	*N* = 149 9 ± 3	*N* = 331 9 ± 3
Cardiac index (L/min/m^2^)	*N* = 73 2.5 ± 0.7	*N* = 176 2.4 ± 0.6	*N* = 79 2.6 ± 0.8	*N* = 164 2.4 ± 0.6	*N* = 38 2.7 ± 0.7	*N* = 87 2.3 ± 0.6	*N* = 41 2.5 ± 0.9	*N* = 77 2.4 ± 0.7	*N* = 152 2.5 ± 0.8	*N* = 340 2.4 ± 0.6
Pulmonary vascular resistance, ^c^ (dyne/sec/cm^5^)	*N* = 76 740 ± 370	*N* = 177 860 ± 500	*N* = 79 690 ± 320	*N* = 168 890 ± 420	*N* = 38 690 ± 240	*N* = 88 920 ± 420	*N* = 41 700 ± 350	*N* = 80 850 ± 430	*N* = 155 720 ± 340	*N* = 345 870 ± 460

*FC* Functional class, *PAH* Pulmonary arterial hypertension, *SD* Standard deviation, WHO World Health Organization

^a^Post-hoc summary; ^b^North America (vs. Rest of World, mostly Western Europe), ^c^Values have been rounded to two significant digits for ease of comparison

### Primary endpoint

**Fig. 3:**
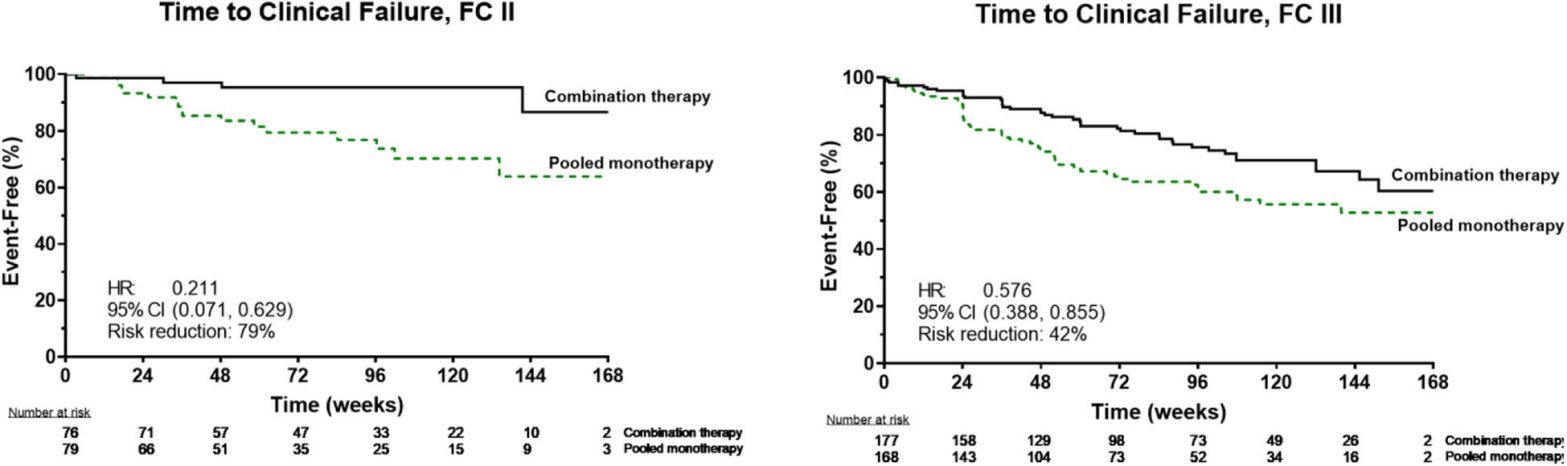
Kaplan-Meier Curves Showing the Probability of First Adjudicated Primary Endpoint (Clinical Failure) Event by Treatment Group and Functional Class

**Fig. 4:**
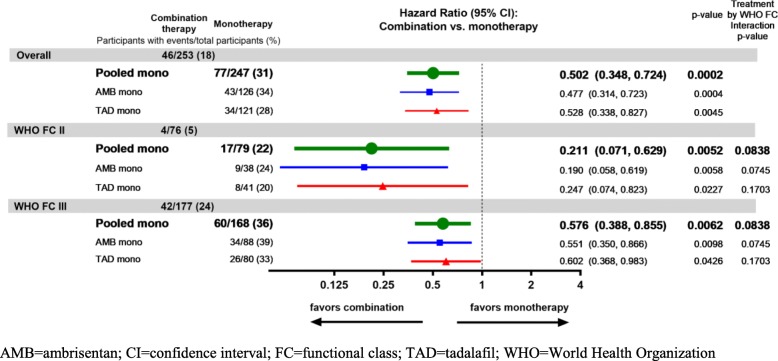
Forest Plot of Time to First Clinical Failure Event by WHO Functional Class and Treatment Group (Primary Analysis Set)

The Kaplan-Meier representation of TtCF comparing combination therapy vs. pooled monotherapy for the subgroups with FC II or III symptoms at baseline is shown in Fig. 3a and b respectively. Figure 3a illustrates the few events in the combination therapy group. The Forest plot of TtCF (Fig. 4) demonstrates that, compared to the results from pooled monotherapy, there is a 50% risk reduction in events for the entire PAS. While participants assigned to initial combination therapy had lower aggregate risk compared to monotherapy, a significantly greater reduction in the risk of clinical events was observed for the FC II cohort (hazard ratio 0.21 [95% CI: 0.07, 0.63]) as compared to the cohort with FC III symptoms (hazard ratio 0.58 [95% CI: 0.39, 0.86]). The statistical analysis for this subgroup interaction suggested that the reduction in clinical failure events with initial combination therapy was more likely in FC II participants than FC III participants (*p* = 0.084, values < 0.1 generally considered significant for this interaction).

Table 3 shows the number of participants experiencing each type of first clinical failure event. PAH-related hospitalizations as a first TtCF event were less likely for participants assigned to initial combination therapy in FC II and FC III participants. In the FC III combination therapy group, 10 (6%) patients had initial PAH-related hospitalizations as their TtCF event as compared to 22 (13%) in the pooled monotherapy group. There were no hospitalizations for PAH as an initial event in FC II participants in the combination therapy group compared to 8 (10%) with hospitalizations for PAH in the monotherapy group.

**Table 3:** Summary of First Clinical Failure Events and First PAH Hospitalizations by WHO Functional Class and Treatment Group (Primary Analysis Set)

	Combination Therapy		Pooled Monotherapy		Ambrisentan Monotherapy		Tadalafil Monotherapy	
	(*n* = 253)		(*n* = 247)		(*n* = 126)		(*n* = 121)	
Baseline Functional Class	FC II	FC III	FC II	FC III	FC II	FC III	FC II	FC III
	(*n* = 76)	(*n* = 177)	(*n* = 79)	(*n* = 168)	(*n* = 38)	(*n* = 88)	(*n* = 41)	(*n* = 80)
First Clinical Failure Events								
Number (%) of patients with event	4 (5)	42 (24)	17 (22)	60 (36)	9 (24)	34 (39)	8 (20)	26 (33)
Death	3 (4)	6 (3)	1 (1)	7 (4)	0	2 (2)	1 (2)	5 (6)
PAH Hospitalization	0	10 (6)	8 (10)	22 (13)	5 (13)	13 (15)	3 (7)	9 (11)
Disease Progression	0	10 (6)	5 (6)	11 (7)	3 (8)	9 (10)	2 (5)	2 (3)
ULTCR	1 (1)	16 (9)	3 (4)	20 (12)	1 (3)	10 (11)	2 (5)	10 (13)
Hazard ratio (95% CI) combination vs.			0.211 (0.071,0.629)	0.576 (0.388,0.855)	0.190 (0.058,0.619)	0.551 (0.350,0.866)	0.247 (0.074,0.823)	0.602 (0.368,0.983)
First PAH Hospitalizations^a^								
Number (%) of patients with event	0	19 (11)	11 (14)	33 (20)	7 (18)	20 (23)	4 (10)	13 (16)
Hazard ratio (95% CI) combination vs.			N/A	0.484 (0.275,0.852)	N/A	0.435 (0.232,0.815)	N/A	0.554 (0.273,1.124)

*CI* Confidence interval, *FC* Functional class, *N/A* Not applicable, *PAH* Pulmonary arterial hypertension, *ULTCR* Unsatisfactory long-term clinical response, *WHO* World Health Organization

^a^Post-hoc analysis

Additional PAH-related hospitalizations occurred after the first clinical failure event. Post-hoc analyses demonstrated that among FC III participants randomized to combination therapy, 19 (11%) had a PAH-related hospitalization over the course of the study compared to 33 (20%) of those in the pooled monotherapy group (Table 3). In contrast, among the FC II participants randomized to combination therapy, there were no PAH-related hospitalizations during the study compared to 11 (14%) in those with FC II symptoms assigned to monotherapy. At baseline, those 11 participants with FC II symptoms had modestly higher NT-pro BNP levels and lower 6MWD, but the distribution of disease severity appeared similar to the entire FC II group (see Additional file 1).

### Secondary endpoints

**Fig. 5:**
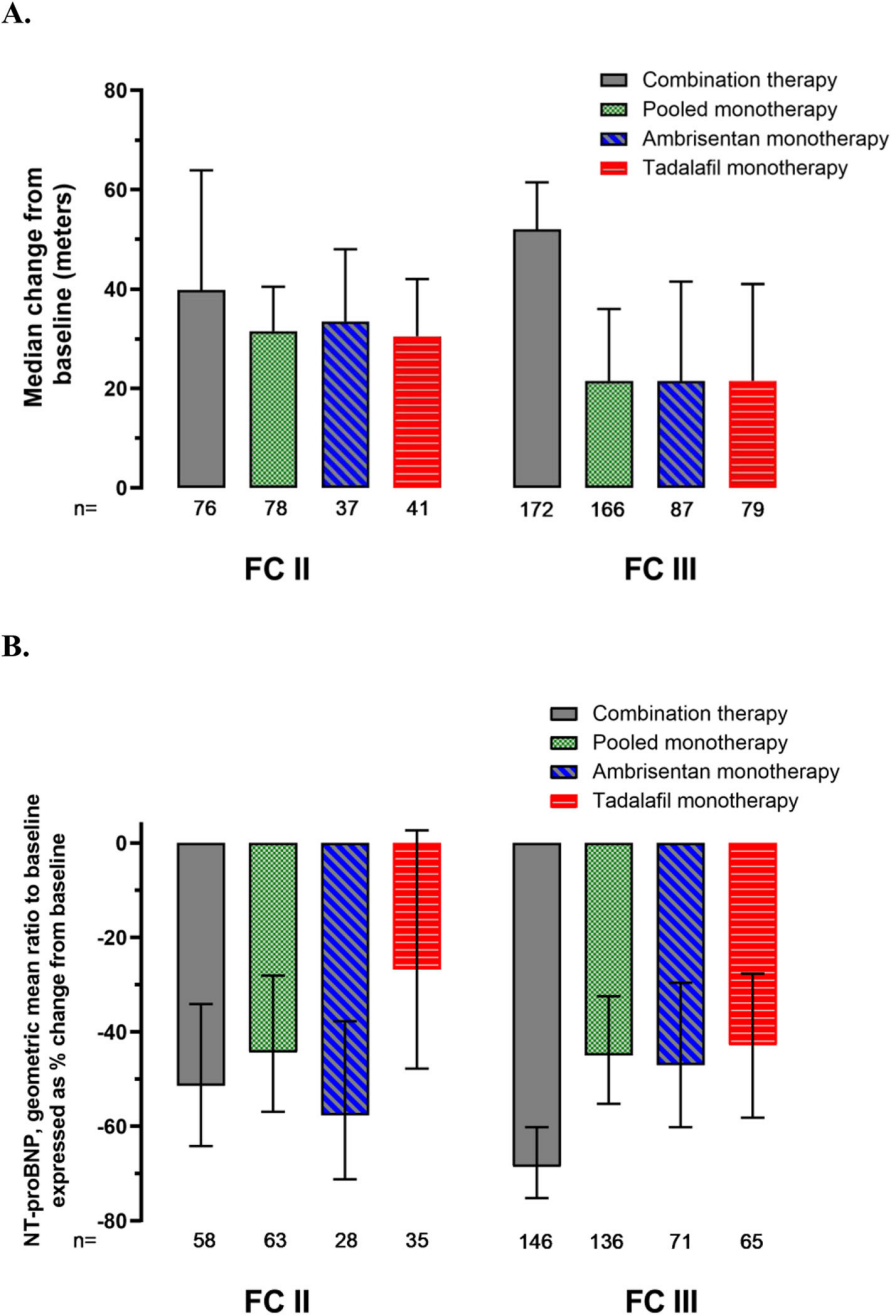
Change in 6-Minute Walk Distance and NT-proBNP from Baseline at Week 24 by WHO Functional Class and Treatment Group (Primary Analysis Set). **a** 6 Minute Walk. Error bars represent 95% confidence intervals. Stratified Wilcoxon Rank Sum analysis: worst rank scores were used for missing data following death or adjudicated hospitalization; otherwise, last observation carried forward imputation was used. FC = functional class; WHO=World Health Organization. **b** NT-pro BNP. Error bars represent 95% confidence intervals. Geometric mean ratio estimates from Mixed Models Repeated Measures analysis adjusted for randomization stratification factors and baseline value, with no imputation for missing data. Terms in model: Treatment, Baseline value, Visit, Treatment x Visit, Etiology of PAH (stratification value) and WHO functional class (stratification value). Stratification values may differ from baseline subgroup values. This is a post-hoc analysis

Participants with FC III symptoms assigned to combination therapy enjoyed a larger treatment effect in 6MWD at Week 24 as compared to pooled monotherapy (Fig. 5a, 52 m vs. 22 m median increase; *p* < 0.001). The difference between treatment groups for improvement in walk was not significant for those with baseline FC II symptoms (40 m vs. 32 m median increase, *p* = 0.366).

Fifty-three percent (53%) of FC II participants randomized to combination therapy demonstrated a satisfactory clinical response (defined as a ≥ 10% improvement in 6MWD over baseline, FC I or II symptoms at Week 24, and no event of clinical worsening through week 24), compared to 43% of the pooled monotherapy group (*p* = 0.243). Among FC III participants, corresponding response rates were 33 and 23%, respectively (*p* = 0.044).

A post-hoc analysis demonstrated that NT-proBNP levels dropped from baseline to Week 24 in both FC II and III participants (Fig. 5b). NT-proBNP fell more among FC III participants randomized to combination therapy vs. pooled monotherapy (geometric mean ratio expressed as difference, − 43% [95% CI: − 54, − 29], *p* < 0.001). Reductions in NT-proBNP for FC II participants were comparable between the two treatment groups (*p* = 0.380).

### Safety

In both functional classes, adverse events were generally more frequent in the combination therapy vs. monotherapy groups for the events listed in Table 4. For the common events of headache, nasal congestion, and nasopharyngitis, less symptomatic FC II participants reported more of these events than FC III participants when assigned to combination therapy. Edema was more often reported for participants randomized to combination (45%) than monotherapy (28–33%), but this did not vary between the functional classes. Therapy discontinuations for adverse events were unusual but more common in those with FC III symptoms; discontinuations did not differ between treatment assignments. Seven FC II patients (9%) on combination therapy and 8 FC II patients (10%) on monotherapy discontinued treatment due to adverse events compared to 29 FC III patients (16%) on combination therapy and 34 FC III patients (20%) on monotherapy.

**Table 4:** Most Frequently (≥10% of Patients) Reported Adverse Events Occurring with Higher Frequency in the Combination Therapy Group (≥5% Difference Between Combination Group and Either Monotherapy Group) in Either WHO Functional Class II or III by Treatment Group

Adverse Event, *n* (%)^a^	Combination Therapy (*n* = 253)		Ambrisentan Monotherapy (*n* = 126)		Tadalafil Monotherapy (*n* = 121)	
	FC II (*n* = 76)	FC III (*n* = 177)	FC II (*n* = 38)	FC III (*n* = 88)	FC II (*n* = 41)	FC III (*n* = 80)
Any event	74 (97)	173 (98)	35 (92)	85 (97)	38 (93)	76 (95)
Headache	39 (51)	68 (38)	8 (21)	33 (38)	14 (34)	28 (35)
Edema peripheral	34 (45)	81 (46)	12 (32)	29 (33)	12 (29)	22 (28)
Nasal congestion	22 (29)	32 (18)	8 (21)	11 (13)	5 (12)	10 (13)
Nasopharyngitis	15 (20)	22 (12)	6 (16)	20 (23)	6 (15)	12 (15)
Cough	13 (17)	27 (15)	5 (13)	9 (10)	7 (17)	14 (18)
Dizziness	13 (17)	37 (21)	6 (16)	18 (20)	4 (10)	10 (13)
Pain in extremity	13 (17)	24 (14)	4 (11)	10 (11)	4 (10)	14 (18)
Flushing	12 (16)	26 (15)	5 (13)	13 (15)	4 (10)	7 (9)
Non-cardiac chest pain	11 (14)	16 (9)	2 (5)	8 (9)	2 (5)	6 (8)
Vomiting	11 (14)	17 (10)	3 (8)	8 (9)	3 (7)	9 (11)
Palpitations	10 (13)	18 (10)	5 (13)	15 (17)	3 (7)	14 (18)
Anemia	9 (12)	28 (16)	1 (3)	7 (8)	5 (12)	9 (11)
Bronchitis	8 (11)	19 (11)	1 (3)	4 (5)	4 (10)	6 (8)
Epistaxis	8 (11)	14 (8)	1 (3)	4 (5)	4 (10)	7 (9)
Dyspepsia	6 (8)	23 (13)	0	5 (6)	6 (15)	8 (10)

*FC* Functional class, *WHO* World Health Organization

^a^Includes adverse events with onset between the first dose of study drug and last dose + 30 days

## Discussion

Initial combination therapy with ambrisentan and tadalafil reduced the risk of clinical events by 50% as compared to those assigned monotherapy, an effect driven by a substantial reduction in hospitalizations. Previous studies have generally found the largest treatment effects in participants with more advanced symptoms at enrollment, and we anticipated similar results. Instead, for the primary endpoint of clinical worsening, the benefit favoring initial combination therapy was numerically larger for participants with FC II symptoms at baseline. There were no PAH-related hospitalizations in FC II participants assigned to combination therapy.

This is the first controlled study of initial combination therapy in PAH, and our hypotheses were driven by older trials of monotherapy and more recent studies of sequential combination therapy. Benefits in exercise tolerance tended to be greater for participants with more advanced symptoms in the registration trials for subcutaneous treprostinil [8] and sildenafil [9]. In recent sequential combination therapy studies for riociguat [5] and macitentan [4], exercise tolerance benefits were similarly muted in FC II participants as compared to those in FC III. .

In the present study, while the overall hospitalization and TtCF event rates were lower in the FC II vs. FC III subgroups, the treatment impact of combination therapy in the FC II participants was numerically greater as compared to those with FC III symptoms (Fig. 4). Moreover, over a median treatment exposure of 76 weeks, there were no PAH-related hospitalizations (nor initiation of parenteral prostacyclin) in the FC II combination therapy cohort compared to 11 PAH-related hospitalizations (14%) in the FC II monotherapy cohort (median exposure 69 weeks in pooled monotherapy). Importantly, this analysis of AMBITION argues that despite having FC II symptoms, treatment naïve patients are still at risk for events including costly hospitalization. Many of the FC II participants had intermediate or high-risk elevations in NT-pro-BNP, emphasizing the importance of a multi-faceted risk evaluation as recently recommended [10, 11]. One caution: this is the primary analysis set of the AMBITION data, excluding those with excess morbidities which suggest left heart disease. The results might not apply to a group with comorbidities typical of an older Western population.

Even considering a multi-modal risk-assessment strategy [12, 13], the 11 FC II participants who had PAH-related hospitalizations on monotherapy did not appear to be the ‘sickest’ of the FC II participants (see details in Additional file 1). This observation suggests that predicting hospitalization among FC II patients is difficult and supports the use of initial combination therapy to decrease the risk of hospitalization. Using the REVEAL score to perform a baseline risk stratification, we have recently reported that initial combination therapy reduces events even in those at lowest baseline risk; in fact, similar to the present results, low REVEAL risk participants had zero events when assigned to combination therapy (as compared to 16% of low risk participants assigned to monotherapy) [10]. The present analysis demonstrates that this is true for all PAH related hospitalizations, not just those occurring as the first clinical failure event.

This analysis stands in contrast to the other two positive, event-driven studies recently completed. The majority of the participants in the studies for macitentan [4] and selexipag [6] were on background therapy at study entry. The point estimate of the treatment effect (risk reduction for clinical events) was similar in the FC II vs. FC III/IV participants for both studies, but the statistical significance of the treatment effect was driven by the FC III/IV participants. The data in Fig. 4 suggest a numerically greater treatment effect for FC II participants in AMBITION, and the statistical analysis for this subgroup interaction suggests that indeed FC II participants had greater benefit. Obviously, GRIPHON and SERAPHIN are very different in design from the present one which focused on treatment-naïve individuals, and even given the different designs, a subset analysis of the GRIPHON data did suggest benefit for FC II participants already on two therapies [14]. One speculative explanation for our data is that treatment naïve patients with FC II symptoms present a unique opportunity to change the disease trajectory if initially treated with an endothelin receptor antagonist (ambrisentan) and a phosphodiesterase inhibitor (tadalafil).

Similar to previously published data (and in contrast with the primary endpoint data), improvements in walk distance and reductions in NT-proBNP for combination therapy vs. monotherapy were muted for patients with FC II symptoms as compared to those in FC III. This is entirely compatible with results from SERAPHIN and is probably easiest understood in terms of the quantitative aspects of analyzing continuous variables. Baseline 6MWD was lower and baseline NT-proBNP values were higher for those in FC III as expected, and the number of participants in FC II was less than half that in FC III. These factors reduced the likelihood of finding a statistically significant difference for functional parameters within FC II participants, although the 6MWD did improve by 40 m in the combination therapy group (for context, a cohort of FC II participants treated with bosentan had an 11 m improvement [15]). There was an absolute 10% increase in the number of participants who achieved a ‘satisfactory clinical response’ for FC II participants treated with combination ambrisentan and tadalafil (vs. pooled monotherapy), and this was identical to the absolute 10% increase in FC III participants with combination therapy. CTD-PAH participants were more likely to be FC III as opposed to FC II but this difference was parallel in combination and pooled monotherapy assigned participants.

Nearly all participants reported some adverse effect, but for the most frequently reported adverse events, FC II participants assigned to combination therapy reported more events than those in FC III. This observation, however, did not translate into a greater rate of drug discontinuation, which was in fact higher among FC III participants. Presumably, participants who were less symptomatic from PAH were more likely to report headache and nasopharyngeal congestion but ultimately acclimated in the context of clinical and functional benefit. Edema, while more common in those assigned to combination therapy, was equally prevalent in the two functional classes and rarely a cause for drug discontinuation.

## Conclusions

In summary, initial combination therapy with ambrisentan and tadalafil reduced clinical failure events, principally PAH-related hospitalizations, among those with FC II symptoms at baseline. FC III participants assigned to combination therapy also had fewer clinical failure events, including PAH-related hospitalization; however, in contrast to previously published data, the magnitude of risk reduction for the primary outcome was numerically greater in those with FC II symptoms at enrollment. Because the combination was well tolerated and safe, this data supports recently amended treatment guidelines suggesting initial combination therapy, particularly for patients with FC II symptoms. Clinicians should find this data valuable in their treatment of newly diagnosed patients with PAH even when symptoms are relatively mild.

## Supplementary information

Additional file 1: Additional method/statistical details and list of investigators. (DOCX 68 kb)

## Data Availability

GSK makes available anonymized individual participant data and associated documents from interventional clinical studies which evaluate medicines, upon approval of proposals submitted to <http://www.clinicalstudydatarequest.com>. To access data for other types of GSK sponsored research, for study documents without patient-level data and for clinical studies not listed, please submit an enquiry via the website.

## Abbreviations

FC: Functional class
NT-proBNP: N-terminal pro-B-type natriuretic peptide
PAH: Pulmonary arterial hypertension
PAS: Primary analysis set
TtCF: Time to first clinical failure
