# Supplementary material for: Clinical outcomes stratified by baseline functional class after initial combination therapy for pulmonary arterial hypertension
Source: Respir Res. 2019 Sep 12;20:208. doi: 10.1186/s12931-019-1180-1 (PMC6739949; doi:10.1186/s12931-019-1180-1)
Supplement: Supplementary file 1 — Additional method/statistical details and list of investigators. (DOCX 68 kb) [file 12931_2019_1180_MOESM1_ESM.docx]

**SUPPLEMENTAL MATERIAL**

**Clinical Outcomes Stratified by Baseline Functional Class After Initial Combination Therapy for Pulmonary Arterial Hypertension**

R. James White, M.D., Ph.D., Anton Vonk-Noordegraaf, M.D., Stephan Rosencranz, M.D., Ronald J. Oudiz, M.D., Vallerie V. McLaughlin, M.D., Marius M. Hoeper, M.D., Ekkehard Grünig, M.D., Hossein-Ardeschir Ghofrani, M.D., Murali Chakinala, M.D., Joan A. Barberà, M.D., Christiana Blair, M.S., Jonathan Langley, B.Sc., Adaani E. Frost, M.D.

**List of Investigators and IECs/IRBs for AMBITION (NCT01178073; EudraCT Number: 2009-011150-17)**

| **Investigator** | **Sub-Investigator** | **Description of Research**  **Facility, Hospital/ Institution, and Address** | **Name of IEC/IRB Committee,**  **Address, Committee Chair** |
| --- | --- | --- | --- |
| **Australia** |  |  |  |
| Corte, Tamera. PhD  Celermajer, David. MBBS, PhD (FPI) Youssef, Peter Paul. MBBS, FRACP(FPI) | Lau, Edmund | Royal Prince Alfred Hospital,  Respiratory Medicine Department, Missenden Road, Camperdown, New South Wales, 2050, Australia | Sydney Local Health District Ethics  Review Committee (RPAH Zone), Research Development Office, Level 3, Building 92, Royal Prince Alfred Hospital, Missenden Road, Camperdown, 2050, Australia  Chairperson: Loblay, Robert |
| Keogh, Anne. MD,  FRACP | Grover, Rominder S  Kotlyer, Eugene  Lim, Choon P  MacDonald, Peter  Spina, Roberto | St Vincents Hospital, Heart Lung Transplant Clinic, Level 4, Xavier Building, 390 Victoria Street, Darlinghurst, New South Wales, 2010, Australia | Sydney Local Health District Ethics  Review Committee (RPAH Zone), Research Development Office, Level 3, Building 92, Royal Prince Alfred Hospital, Missenden Road, Camperdown, 2050, Australia  Chairperson: Loblay, Robert |
| Kermeen, Fiona D.  MBBS, FRACP | Chambers, Daniel  Hopkins, Peter | Prince Charles Hospital, Thoracic Admin Building, Level 1, Rode Road, Chermside, Queensland, 4032, Australia | The Prince Charles Hospital Northside Health Service District HREC, Rode Road, Chermside, Queensland, 4032, Australia  Chairperson: Denman, Russell |
| Kilpatrick, David. MD, FRACP* | Dwyer, Nathan. PhD | Royal Hobart Hospital, 48  Liverpool Street, Hobart, Tasmania, 7000, Australia | Human Research Ethics Committee (Tasmania) Network, University of Tasmania, Private Bag 01, Hobart, Tasmania, Australia  Chairperson: Cowen-Kitanovic, Rachel |
| Lavender, Melanie A. MBChB, MRCP* | Gabbay, Eli | Royal Perth Hospital, Advanced Lung Disease Clinic, Level 3 Ainslie House, Wellington Street, Perth, Western Australia, 6000, Australia | Royal Perth Hospital Ethics Committee, Kirkman House, Wellington Street Campus, Perth, Western Australia, 6847, Australia  Chairperson: Van Bockxmeer, Frank |
| Williams, Trevor J.  MD, FRACP* | Keating, Dominic  Whitford, Helen  Wrobel, Jeremy | The Alfred Hospital, Department of Allergy Immunology & Respiratory Medicine, 5th Floor, Main Ward Block, Commercial Road, Melbourne, Victoria, 3004, Australia | The Alfred Hospital, Human Research Ethics Committee, Commercial Road, Melbourne, Victoria, 3004, Australia  Chairperson: McNeil, John |
| **Austria** |  |  |  |
| Kaehler, Christian.  MD | Cima, Katharina  Desole, Susanna  Loeffler-Ragg, Judith  Vogelsinger, Helene | Medizinische Universität  Innsbruck, Universitätsklinik für Innere Medizin VI, Anichstrasse 35, Innsbruck, 6020, Austria | EK der Med. Universität Wien,  Borschkegasse 8b/E06, Vienna, A-1090, Austria  Chairperson: Singer, Ernst |
| Lang, Irene M. Dr.  med | Renner, Maria  Sadushi-Kolici, Roela  Skoro Sajer, Nika | Univ. Clinic for Internal Medicine II / Cardiology, Waehringer Guertel 18-20, Vienna, 1090, Austria | EK der Med. Universität Wien,  Borschkegasse 8b/E06, Vienna, A-1090, Austria  Chairperson: Singer, Ernst |
| **Belgium** |  |  |  |
| Delcroix, Marion. MD, PhD | Belge, Catharina. MD  Wuyts, Wim. MD | Universitair Ziekenhuis Gasthuisberg, Department of Pneumology, Here Street 49, Leuven, 3000, Belgium | Cliniques Universitaires de Bruxelles - Hôpital Erasme, Comité d'Ethique, Route de Lennik 808, Brussels, 1070, Belgium  Chairperson: Herchuelz, André |
| Vachiery, Jean-Luc E. MD | Huez, Sandrine. MD  Naeije, Robert. MD, PhD  Yerly, Patrick. MD | Cliniques Universitaires de  Bruxelles - Hôpital Erasme, Department of Cardiology, Route de Lennik, 808, Bruxelles, 1070, Belgium | Cliniques Universitaires de Bruxelles - Hôpital Erasme, Comité d'Ethique, Route de Lennik 808, Brussels, 1070, Belgium  Chairperson: Herchuelz, André |
| **Canada** |  |  |  |
| Bshouty, Zoheir. DM, PhD | Ramsey, Clare | Health Sciences Centre,  Respiratory Hospital, RS317-810 Sherbrook Street, Winnipeg, Manitoba, R3A 1R8, Canada | University of Manitoba, Biomedical  Ethics Research Board, P123, 770  Bannatyne Avenue, Winnipeg, Manitoba, R3E 0W3, Canada  Chairperson: Nicolle, Lindsay |
| Granton, John. MD,  FRCPC | Moric, Jakov. MD  Thenganatt, John. MD | Toronto General Hospital, 11C-1170 CSB, 585 University Avenue, Toronto, Ontario, M5G 2N2, Canada | Research Ethics Board, 10th floor room 10-56, 700 University Avenue, Toronto, Ontario, M5G 1Z5, Canada  Chairperson: Hesslegrave, Ronald |
| Hirani, Naushad. MD, FRCPC | Fisher, Dina. MD  Helmersen, Douglas. MD Loewen, Andrea. MD Rimmer, Karen P. MD Thakrar, Mitesh V. MD, FRCPC  Viner, Sid M | Peter Lougheed Center, Respiratory Research, Room 1100, 3500 26 Avenue North East, Calgary, Alberta, T1Y 6J4, Canada | University of Calgary - Conjoint Health Research Ethics Board, 3rd Floor, Mackimmie Library Tower (MLT 300), 2500 University Drive NW, Calgary, Alberta, T2N 1N4, Canada  Chairperson: Page, Stacey |
| Provencher, Steeve.  MD, FRCPC | Dion, Geneviève. MD  Martel, Simon. MD  Simon, Mathieu. MD | Institut Universitaire de Cardiologie et de Pneumologie de Québec, 2725 Chemin Sainte Foy, Quebec City, Québec, G1V 4G5, Canada | Institut Universitaire de Cardiologie et de Pneumologie de Québec, Comité d'éthique de la recherche, 2725 Chemin Ste-Foy, Quebec, Québec, G1V 4G5, Canada  Chairperson: Molin, Frank |
| Swiston, John R. MD, MPH | Levy, Robert D. MD | Vancouver General Hospital, The Lung Centre, 7th Floor, 2775 Laurel Street, Vancouver, British Columbia, V5Z 1M9, Canada | University of British Columbia, Clinical Research Ethics Board, Room 210, 828 West 10th Avenue, Vancouver, British Columbia, V5Z 1L8, Canada  Chairperson: Bellward, Gail |
| **France** |  |  |  |
| Bourdin, Arnaud. MD, PhD | Chiron, Raphaël. MD  Meziane, Lahouari. MD | CHRU de Montpellier – Hôpital Arnaud de Villeneuve, Service des Maladies Respiratoires, 371, avenue du Doyen Gaston Giraud, Montpellier cedex 5, 34295,  France | CPP Ile de France VII - Hôpital Bicêtre, Secteur Marron - Pavillon La Force / Porte 58 - 1er étage, 78, rue du Général Leclerc, Le Kremlin Bicêtre cedex, 94275, France  Chairperson: Carre, Jacques |
| Chabot, Jean-  François. MD | Chaouat, Ari. MD  Guillaumot, Anne | CHU de Nancy - Hôpital pour  Adultes de Brabois, Service Pneumologie, Tour Drouet, Rue du Morvan, Vandoeuvre-les- Nancy, 54511, France | CPP Ile de France VII - Hôpital Bicêtre, Secteur Marron - Pavillon La Force / Porte 58 - 1er étage, 78, rue du Général Leclerc, Le Kremlin Bicêtre cedex, 94275, France  Chairperson: Carre, Jacques |
| Cottin, Vincent. MD,  PhD | Cordier, Jean-François. MD  Khouatra, Chahera. MD  Kiakouama-Maleka, Lize.  MD  Piegay, Fabrice. MD Traclet, Julie | Hôpital Louis Pradel, Service  Pneumologie, Centre de Référence des Maladies Pulmonaires Rares, 28, avenue  Doyen Lépine, Lyon Cedex, Bron, 69677, France | CPP Ile de France VII - Hôpital Bicêtre, Secteur Marron - Pavillon La Force / Porte 58 - 1er étage, 78, rue du Général Leclerc, Le Kremlin Bicêtre cedex, 94275, France  Chairperson: Carre, Jacques |
| De Groote, Pascal.  MD | Fertin, Marie. MD  Hachulla, Eric. MD  Lambert, Marc. MD  Launay, David. MD | CHRU de Lille – Hôpital Cardiologique, Cardiologie, Boulevard du Pr Jules Leclercq, Lille, 59037, France | CPP Ile de France VII - Hôpital Bicêtre, Secteur Marron - Pavillon La Force / Porte 58 - 1er étage, 78, rue du Général Leclerc, Le Kremlin Bicêtre cedex, 94275, France  Chairperson: Carre, Jacques |
| Dromer, Claire. MD | Blanchard, Elodie. MD  Nubret-Le-Coniat, Karine. MD  Picard, François. MD | CHU de Bordeaux - Hôpital Haut-Lévêque, G3 - Unité Pneumologie, Bâtiment du Centre François Magendie, Avenue de Magellan, Pessac cedex, 33604, France | CPP Ile de France VII - Hôpital Bicêtre, Secteur Marron - Pavillon La Force / Porte 58 - 1er étage, 78, rue du Général Leclerc, Le Kremlin Bicêtre cedex, 94275, France  Chairperson: Carre, Jacques |
| Frachon, Irène. MD* | Barnier, Aude. MD  Couturaud, Francis. MD  Gut-Gobert, Christophe. MD  Le Henaff, Mikael. MD Leroyer, Christophe. MD | CHU de Brest - Hôpital de la Cavale Blanche, Service Pneumologie 2, Boulevard Tanguy Prigent, Brest Cedex,  29609, France | CPP Ile de France VII - Hôpital Bicêtre, Secteur Marron - Pavillon La Force / Porte 58 - 1er étage, 78, rue du Général Leclerc, Le Kremlin Bicêtre cedex, 94275, France  Chairperson: Carre, Jacques |
| Paganin, Fabrice. MD | Huchot, Eric. MD  Peyrat, Elsa. MD  Poubeau, Patrice. MD | CHU la Réunion, GHSR-Service Pneumologie et Maladies Infectieuses, Médecine R, Avenue du Président Mitterrand, Saint Pierre cedex, 97448, France | CPP Ile de France VII - Hôpital Bicêtre, Secteur Marron - Pavillon La Force / Porte 58 - 1er étage, 78, rue du Général Leclerc, Le Kremlin Bicêtre cedex, 94275, France  Chairperson: Carre, Jacques |
| Pison, Christophe.  MD | Camara, Boubou. MD  Quetant, Sébastien. MD  Saint-Raymond, Christel. MD | CHU de Grenoble - Hôpital Albert Michallon, Service Pneumologie, Boulevard de la Chantourne, La Tronche, 38700, France | CPP Ile de France VII - Hôpital Bicêtre, Secteur Marron - Pavillon La Force / Porte 58 - 1er étage, 78, rue du Général Leclerc, Le Kremlin Bicêtre cedex, 94275, France  Chairperson: Carre, Jacques |
| Reynaud-Gaubert,  Martine. MD | Coltey, Berengere. MD  Dufeu, Nadine. MD  Nieves, Ana. MD | CHU de Marseille - Hôpital Nord, Service de Pneumologie, Chemin des Bourrely, Marseille cedex 20, 13915, France | CPP Ile de France VII - Hôpital Bicêtre, Secteur Marron - Pavillon La Force / Porte 58 - 1er étage, 78, rue du Général Leclerc, Le Kremlin Bicêtre cedex, 94275, France  Chairperson: Carre, Jacques |
| Simonneau, Gérald.  MD | Jais, Xavier. MD  Montani, David. MD Parent, Florence  Savale, Laurent. MD  Sitbon, Olivier. MD | Hôpital Kremlin Bicêtre, Service de Pneumologie et Réanimation Respiratoire, Pavillon BROCA, 78, rue du Général Leclerc, Le Kremlin-Bicêtre cedex, 94275, France | CPP Ile de France VII - Hôpital Bicêtre, Secteur Marron - Pavillon La Force / Porte 58 - 1er étage, 78, rue du Général Leclerc, Le Kremlin Bicêtre cedex, 94275, France  Chairperson: Carre, Jacques |
| Tetu, Laurent. MD | Prevot, Grégoire. MD | CHU de Toulouse - Hôpital Larrey, Service Pneumologie - Pôle Voies Respiratoires, 24, chemin de Pouvourville - TSA 30030, Toulouse cedex 9, 31059, France | CPP Ile de France VII - Hôpital Bicêtre, Secteur Marron - Pavillon La Force / Porte 58 - 1er étage, 78, rue du Général Leclerc, Le Kremlin Bicêtre cedex, 94275, France  Chairperson: Carre, Jacques |
| **Germany** |  |  |  |
| Ewert, Ralf. Dr. med | Bollmann, Tom. Dr. med | Universitaetsklinikum Greifswald, Department of Internal Medicine, Respiratory Medicine and Infectious Diseases, Friedrich- Loeffler-Strasse 23a, Greifswald, Mecklenburg-Vorpommern, 17487, Germany | Ethikkommission der Medizinischen  Hochschule Hannover, Carl-Neuberg- Strasse 1, Hannover, Niedersachsen, 30625, Germany  Chairperson: Troeger, Hans-Dieter |
| Ghofrani, Hossein Ardeschir. Dr. med | Belly, Michael  Jan, Grimminger  Karadas, Burcu  Milger, Katrin. Dr. med Schweitzer, Jassin Sommer, Natascha. Dr. med  Sommerlad, Janine  Thamm, Melanie  Tiede, Henning. Dr. med  Vosswinckel, Robert | Universitaetsklinikum Giessen,  Klinik Strasse 33, Giessen, Hessen, 35392, Germany | Ethikkommission der Medizinischen Hochschule Hannover, Carl-Neuberg- Strasse 1, Hannover, Niedersachsen,  30625, Germany  Chairperson: Troeger, Hans-Dieter |
| Gruenig, Ekkehard.  Dr. med | Benz, Andreas. Dr. med  Corvinus, Christoph. Dr. med  Egenlauf, Benjamin. Dr. med  Lichtblau, Mona. Dr. med  Nagel, Christian. Dr. med | Thoraxklinik Heidelberg gGmbH, Amalien Strasse 5, Heidelberg, Baden-Wuerttemberg, D-69126, Germany | Ethikkommission der Medizinischen Hochschule Hannover, Carl-Neuberg- Strasse 1, Hannover, Niedersachsen,  30625, Germany  Chairperson: Troeger, Hans-Dieter |
| Held, Matthias. Dr.  med | Gunreben, Johanna. Dr.  med  Holl, Regina. Dr. med Romen, Tobias. Dr. med  Schroeder, Barbara. Dr. med  Walter, Franziska. Dr. med | Missionsaerztliche Klinik Würzburg GmbH Innere Medizin Pneumologie, Salvator Strasse 7, Wuerzburg, Bayern, 97074, Germany | Ethikkommission der Medizinischen Hochschule Hannover, Carl-Neuberg- Strasse 1, Hannover, Niedersachsen,  30625, Germany  Chairperson: Troeger, Hans-Dieter |
| Hoeffken, Gert. Dr.  med | Braun, Silke Doris. Dr. med  Halank, Michael. Dr. med  Kulka, Christine  Schulte-Hubbert, Bernhard. Dr. med | Universitaetsklinikum Carl Gustav Carus Dresden, Medizinische Klinik und Poliklinik I, Fetscher Strasse 74, Dresden, Sachsen,  1307, Germany | Ethikkommission der Medizinischen Hochschule Hannover, Carl-Neuberg- Strasse 1, Hannover, Niedersachsen,  30625, Germany  Chairperson: Troeger, Hans-Dieter |
| Hoeper, Marius M. Dr. med | Golpon, Heiko. Dr. med  Knudsen, Lars. Dr. med Meyer, Katrin. Dr. med Olsson, Karen. Dr. med Rademacher, Jessica. Dr. med | Medizinische Hochschule Hannover, Abteilung Pneumologie, Carl-Neuberg- Strasse 1, Hannover, Niedersachsen, 30625, Germany | Ethikkommission der Medizinischen Hochschule Hannover, Carl-Neuberg- Strasse 1, Hannover, Niedersachsen,  30625, Germany  Chairperson: Troeger, Hans-Dieter |
| Klose, Hans FE. Dr.  med | Anna, Nolde. Dr. med.  Baumann, Hans Joerg. Dr. med  Grimme, Ina. Dr. med  Harbaum, Lars  Hennigs, Jan Kristoff. Dr. med  Heyckendorf, Jan. Dr. med  Oqueka, Tim. Dr. med Strassburg, Mareike | Universitaetsklinikum Eppendorf, Medical Clinic, Pnemologie, Martini Strasse 52, Hamburg, Hamburg, 20246, Germany | Ethikkommission der Medizinischen Hochschule Hannover, Carl-Neuberg- Strasse 1, Hannover, Niedersachsen,  30625, Germany  Chairperson: Troeger, Hans-Dieter |
| Lange, Tobias J. Dr.  med  Pfeifer, Michael W (FPI) | Regotta, Sabine Friederike. Dr. med  Schulz, Christian. Dr. med | Universitaetsklinikum Regensburg, Division of Pulmonology, Franz-Josef- Strauss-Allee 11, Regensburg, Bayern, 93053, Germany | Ethikkommission der Medizinischen Hochschule Hannover, Carl-Neuberg- Strasse 1, Hannover, Niedersachsen,  30625, Germany  Chairperson: Troeger, Hans-Dieter |
| Neurohr, Claus. Dr.  med | Baezner, Carlos. Dr. med  Meis, Tobias  Schild, Carolin  Von Wulffen, Werner. Dr. med | Klinikum Grosshadern, Marchionini Strasse 15, Muenchen, Bayern, 81377, Germany | Ethikkommission der Medizinischen Hochschule Hannover, Carl-Neuberg- Strasse 1, Hannover, Niedersachsen,  30625, Germany  Chairperson: Troeger, Hans-Dieter |
| Opitz, Christian. Dr.  med | Zorn, Ruediger. Dr. med | DRK Kliniken Berlin Koepenick, Klinik fuer Innere Medizin Kardiologie, Salvador-Allende- Strasse 2-8, Berlin, Berlin, 12559, Germany | Ethikkommission der Medizinischen Hochschule Hannover, Carl-Neuberg- Strasse 1, Hannover, Niedersachsen,  30625, Germany  Chairperson: Troeger, Hans-Dieter |
| Rosenkranz, Stephan. Dr. med, PhD | Dumitrescu, Daniel. Dr. med  Gerhardt, Felix. Dr. med  Schnitker, Jessika  Viethen, Thomas. Dr. med | Klinikum der Universitaet Koeln, Klinik III fuer Innere Medizin, Kerpener Strasse 62, Koeln, Nordrhein-Westfalen, D-50937, Germany | Ethikkommission der Medizinischen Hochschule Hannover, Carl-Neuberg- Strasse 1, Hannover, Niedersachsen,  30625, Germany  Chairperson: Troeger, Hans-Dieter |
| Skowasch, Dirk. Dr.  med | Byung-Moon, Kim  Pabst, Stefan. Dr. med  Tuleta, Izabela. Dr. med | Universitaetsklinikum Bonn Medizinische Klinik II, Sigmund- Freud-Strasse 25, Bonn, Nordrhein-Westfalen, 53127, Germany | Ethikkommission der Medizinischen Hochschule Hannover, Carl-Neuberg- Strasse 1, Hannover, Niedersachsen,  30625, Germany  Chairperson: Troeger, Hans-Dieter |
| Sorichter, Stephan.  Dr. med | Germann, Martin. Dr. med  Kabitz, Hans-Joachim. Dr. med  Mueller, Tobias. Dr. med Scholz, Tobias. Dr. med | Universitaetsklinikum Freiburg, Department of Internal medicine, Division of Pneumology, Killian Strasse 5, Freiburg, Baden- Wuerttemberg, 79106, Germany | Ethikkommission der Medizinischen Hochschule Hannover, Carl-Neuberg- Strasse 1, Hannover, Niedersachsen,  30625, Germany  Chairperson: Troeger, Hans-Dieter |
| Staehler, Gerd. Dr.  med | Halm, Klaus-Guenther. Dr. med  Volk, Anna. Dr. med | Klinikum Loewenstein GmbH, Medizinische Klinik I, Im Geisshoelzle 62, Loewenstein, Baden-Wuerttemberg, D-74245, Germany | Ethikkommission der Medizinischen Hochschule Hannover, Carl-Neuberg- Strasse 1, Hannover, Niedersachsen,  30625, Germany  Chairperson: Troeger, Hans-Dieter |
| Wilkens, Heinrike. Dr. med | Frantz, Christian  Franzen, Karin | Universitaetsklinikum des Saarlandes, Innere Medizine V, Kirrberger Strasse 100, Homburg, Saarland, 66421, Germany | Ethikkommission der Medizinischen Hochschule Hannover, Carl-Neuberg- Strasse 1, Hannover, Niedersachsen,  30625, Germany  Chairperson: Troeger, Hans-Dieter |
| Wirtz, Hubert RW. Dr. med | Grachtrup, Sabine. Dr. med  Seyfarth, Hans-Juergen. Dr. med | Universitaetsklinikum Leipzig, Liebig Strasse 20, Leipzig, Sachsen, 4103, Germany | Ethikkommission der Medizinischen Hochschule Hannover, Carl-Neuberg- Strasse 1, Hannover, Niedersachsen, 30625, Germany  Chairperson: Troeger, Hans-Dieter |
| **Greece** |  |  |  |
| Anthi, Anastasia. MD | Lekakis, Ioannis. MD, PhD  Orfanos, Stylianos. MD  Triantafyllidi, Helen. MD  Tsagkaris, Iraklis. MD Vrigkou, Eleni. MD, PhD | University General Hospital "ATTIKON", B' Intensive Care Unit-Pulmonary Hypertension Department, 1 Rimini Street, Haidari, Athens, 12462, Greece | National Ethics Committee, 284, Messogion avenue, Athens, 15562, Greece  Chairperson: Papavasileiou, Athanasios |
| Athanasopoulos,  Georgios. MD, PhD | Demerouti, Eftichia. MD | Onassis Cardiac Surgery Center, 1st Department of Cardiology, 356 Syggrou Avenue, Kallithea, Athens, 17674, Greece | National Ethics Committee, 284, Messogion avenue, Athens, 15562, Greece  Chairperson: Papavasileiou, Athanasios |
| Blamis, Kariofillis.  MD* | Papadopoulos, Ioannis. MD, PhD  Sarikoudis, Theodosios. MD | General Hospital of Kavala,  Rheumatology Clinic, Agios Silas, Kavala, 65500, Greece | National Ethics Committee, 284, Messogion avenue, Athens, 15562, Greece  Chairperson: Papavasileiou, Athanasios |
| Bouros, Demosthenes. MD, PhD* | Tzouvelekis, Argyrios. MD, PhD  Xloropoulou, Niki  Zaxaris, Georgios. MD | General University Hospital of Alexandroupolis, Respiratory Clinic, Dragana, Alexandroupolis, 68100, Greece | National Ethics Committee, 284, Messogion avenue, Athens, 15562, Greece  Chairperson: Papavasileiou, Athanasios |
| Georgopoulos,  Dimitrios. MD, PhD* | Kondyli, Eumorfia. MD, PhD  Mitrouska, Ioanna. MD, PhD | University Hospital of Heraklion, Intensive Care Clinic, Stavrakia & Voutes, Heraklion - Crete, 71110, Greece | National Ethics Committee, 284, Messogion avenue, Athens, 15562, Greece  Chairperson: Papavasileiou, Athanasios |
| Konstantinides,  Stavros. MD, PhD* | Chalikias, Georgios. MD  Thomaidi, Adina. MD, PhD | University General Hospital of Alexandroupolis, University Cardiology Clinic, Perioxi Dragana, 68100, Alexandroupolis, 68100, Greece | National Ethics Committee, 284, Messogion avenue, Athens, 15562, Greece  Chairperson: Papavasileiou, Athanasios |
| Stanopoulos, Ioannis.  MD, PhD* | Pitsiou, Georgia. MD, PhD | G. Papanikolaou Hospital, Respiratory Deficiency Unit, Exohi, Thessaloniki, 57010, Greece | National Ethics Committee, 284, Messogion avenue, Athens, 15562, Greece  Chairperson: Papavasileiou, Athanasios |
| Styliadis, Ioannis. MD, PhD | Giannakoulas, George. MD, PhD  Mouratoglou, Sophia- Anastasia. MD  Parcharidou, Despoina. MD | "Ahepa" University General  Hospital of Thessaloniki, A' University Cardiology Clinic, 1 St. Kiriakidis Street, Thessaloniki, 54636, Greece | National Ethics Committee, 284, Messogion avenue, Athens, 15562, Greece  Chairperson: Papavasileiou, Athanasios |
| **Italy** |  |  |  |
| Galiè, Nazzareno. MD | Bachetti, Cristina  Manes, Alessandra Palazzini, Massimiliano Rinaldi, Andrea  Rizzo, Nicole  Sciarra, Francesca  Terzi, Francesca | Azienda Ospedaliero-Universitaria Policlinico S. Orsola Malpighi, Istituto di Cardiologia, Via Massarenti, 9, Bologna, Emilia- Romagna, 40138, Italy | Comitato Etico Indipendente, Via Massarenti, 9, Bologna, 40138, Italy  Chairperson: Montanaro, Nicola |
| Marini, Carlo | Airò, Edoardo  Bauleo, Carolina  Pancani, Roberta | Fondazione Toscana "G. Monasterio" - CNR Regione Toscana, Reparto di Pneumologia, Via Moruzzi n. 1, Pisa, Toscana, 56124, Italy | Comitato Etico Azienda Ospedaliera Universitaria Pisana, Via Roma 67, Pisa, Toscana, 56126, Italy  Chairperson: Danesi, Romano |
| Mulè, Massimiliano  EG. MD  Ussia, Gian Paolo. MD, FSCAI (FPI) | Aruta, Patrizia  Ministeri, Margherita Pistritto, Annamaria Scarabelli, Marilena | Azienda Ospedaliera Universitaria Vittorio Emanuele, Ferrarotto, Santo Bambino, U.O. di Cardiologia, P.O. Ferrarotto Alessi, Via S. Citelli, Catania, Catania, Sicilia, 95100, Italy | Comitato Etico dell’Azienda Ospedaliero Universitaria “Policlinico Vittorio Emanuele”, Via Citelli, Catania, 95124, Italy  Chairperson: Amico-Roxas, Matilde |
| Porcu, Maurizio. MD | Corda, Marco  Giardina, Giorgio  Manca, Ilaria  Orrù, Pierpaolo | Azienda Ospedaliera "G.Brotzu", S.C. Cardiologia-Dip.to Patologia Cardiaca, Piazzale Alessandro Ricchi, 2, Cagliari, Sardegna,  9134, Italy | Comitato Etico Indipendente dell`Azienda Ospedaliera “Giuseppe Brotzu”, Piazzale Alessandro Ricchi, 2, Caliagri, 9134, Italy  Chairperson: Farris, Andreina |
| Vizza, Carmine Dario. MD | Badagliacca, Roberto  Gambardella, Cristina  Nocioni, Martina  Papa, Silvia  Pezzuto, Beatrice  Poscia, Roberto | Policlinico Umberto I – Università La Sapienza, Dipartimento di Scienze Cardiovascolari Respiratorie Morfologiche, Cardiologia, Viale del Policlinico 115, Roma, Lazio, 161, Italy | Comitato Etico dell'Azienda Policlinico Umberto I di Roma, Viale del Policlinico, 155, Roma, Lazio, 161, Italy  Chairperson: Isidori, Aldo |
| **Japan** |  |  |  |
| Abe, Kohtaro. MD,  PhD  Kishi, Takuya. MD, PhD (FPI) | Koike, George. MD, PhD | Kyushu University Hospital, 3-1-1, Maidashi, Higashi-ku, Fukuoka- shi, Fukuoka, 812-8582, Japan | Kyushu University Hospital, 3-1-1, Maidashi, Higashi-ku, Fukuoka-shi, Fukuoka, 812-8582, Japan  Chairperson: Masuda, Satohiro |
| Hatano, Masaru. MD, PhD  Yao, Atsushi. MD, PhD (FPI) | Inaba, Toshiro. MD, PhD  Kinugawa, Koichiro. MD, PhD  Maki, Hisataka. MD, PhD | The University of Tokyo Hospital, 7-3-1, Hongo, Bunkyo-ku, Tokyo, 113-8655, Japan | The University of Tokyo Hospital, 7-3-1, Hongo, Bunkyo-ku, Tokyo, 113-8655, Japan  Chairperson: Sato, Shinichi |
| Tsujino, Ichizo. MD,  PhD* | Ohira, Hiroshi. MD  Takahiro, Sato. MD  Taku, Watanabe. MD, PhD | Hokkaido University Hospital, North 14, West 5, Kita-ku, Sapporo-shi, Hokkaido, 060-8648, Japan | Hokkaido University Hospital, North 14, West 5, Kita-ku, Sapporo-shi, Hokkaido, 060-8648, Japan  Chairperson: Iseki, Ken |
| Watanabe, Hiroshi.  MD, PhD* | Takeuchi, Kazuhiko. MD,  PhD | Hamamatsu University School of Medicine, University Hospital, 1-20-1, Handayama, Higashi-ku, Hamamatsu-city, Shizuoka, 431-3192, Japan | Hamamatsu University School of Medicine, University Hospital, 1-20-1, Handayama, Higashi-ku, Hamamatsu- city, Shizuoka, 431-3192, Japan  Chairperson: Umemura, Kazuo |
| **Netherlands** |  |  |  |
| Boomars, Karin A.  MD, PhD* | Van Empel, Vanessa PM.  MD, PhD | Academisch Ziekenhuis Maastricht, Department of Respiratory Diseases, P. Debyelaan 25, Maastricht, 6229 HX, Netherlands | Vrije Universtiteit Medical Centre, De Boelelaan Meditsch Ethische Toetsingscommissie, 1117, Amsterdam, 1081 HV, Netherlands  Chairperson: Rauwerda, Jan A |
| Van Den Toorn, Leon M. MD, PhD | Boomars, Karin A. MD, PhD  Wijsenbeek-Lourens, Marlies S. MD, PhD | Erasmus MC, 's Gravendijkwal  230, Rotterdam, 3015 CE, Netherlands | Vrije Universtiteit Medical Centre, De Boelelaan Meditsch Ethische Toetsingscommissie, 1117, Amsterdam, 1081 HV, Netherlands  Chairperson: Rauwerda, Jan A |
| Vonk Noordegraaf,  Antonie. MD, PhD | Bogaard, Harm Jan. MD,  PhD  Boonstra, Anko. MD, PhD | Vrije Universtiteit Medical Centre, De Boelelaan 1117, Amsterdam, 1081 HV, Netherlands | Vrije Universtiteit Medical Centre, De Boelelaan Meditsch Ethische Toetsingscommissie, 1117, Amsterdam, 1081 HV, Netherlands  Chairperson: Rauwerda, Jan A |
| **Spain** |  |  |  |
| Barberá Mir, Joan  Albert. MD | Blanco Vich, Isabel. MD  Chamorro Tort, Nuria. MD  Del Pozo Rivas, Roberto.  MD | Hospital Clinic i Provincial de Barcelona, C/ Villarroel, 170, Barcelona, 8036, Spain | Hospital 12 de Octubre, Ctra de Andalucia, Km 5.4 Hospital 12 Octubre, Madrid, 28041, Spain  Chairperson: De Frutos, Javier Ortiz |
| Carrera Lamarca,  Miguel. MD, PhD* | Sala Llinás, Ernest. MD,  PhD | Hospital Universitario Son Espases, Ctra. de Vallmossa, 79, Palma de Mallorca, 7010, Spain | Hospital 12 de Octubre, Ctra de Andalucia, Km 5.4 Hospital 12 Octubre, Madrid, 28041, Spain  Chairperson: De Frutos, Javier Ortiz |
| Del Castillo Palma, Mª Jesús. MD, PhD* | García Hernández,  Francisco José. MD, PhD Sánchez Román, Julio. MD, PhD | Hospital Virgen del Rocío, Avd. Manuel Siurot s/n, Sevilla, 41013, Spain | Hospital 12 de Octubre, Ctra de Andalucia, Km 5.4 Hospital 12 Octubre, Madrid, 28041, Spain  Chairperson: De Frutos, Javier Ortiz |
| Gómez Sánchez,  Miguel Angel. MD, PhD | Barrios Garrido-Lestache,  Elvira. MD, PhD  Del Pozo Rivas, Roberto. MD  Escribano Subias, Pilar.  MD, PhD | Hospital Doce de Octubre, Unidad de lnsuficiencia Cardiaca e Hipertension Pulmonar, Ctra. de Andalucía Km. 5,4, Madrid, 28041, Spain | Hospital 12 de Octubre, Ctra de Andalucia, Km 5.4 Hospital 12 Octubre, Madrid, 28041, Spain  Chairperson: De Frutos, Javier Ortiz |
| Lázaro Salvador,  María. MD, PhD | None | Hospital Virgen de la Salud, Avda. Barber 30, Toledo, 45004, Spain | Hospital 12 de Octubre, Ctra de Andalucia, Km 5.4 Hospital 12 Octubre, Madrid, 28041, Spain  Chairperson: De Frutos, Javier Ortiz |
| López Reyes, Raquel. MD  Nauffal Manzur, Dolores. MD, PhD (FPI) | None | Hospital La Fe, C/ Bulevar Sur s/n, Valencia, 46026, Spain | Hospital 12 de Octubre, Ctra de Andalucia, Km 5.4 Hospital 12 Octubre, Madrid, 28041, Spain  Chairperson: De Frutos, Javier Ortiz |
| Merino Verdugo,  Julio. MD, PhD | Arroyo Varela, Macarena.  MD  De Ramón Garrido, Enrique. MD, PhD Espíldora Hernández, Francisco. MD | Hospital Carlos Haya, Avda, Carlos Haya s/n, Málaga, 29010, Spain | Hospital 12 de Octubre, Ctra de Andalucia, Km 5.4 Hospital 12 Octubre, Madrid, 28041, Spain  Chairperson: De Frutos, Javier Ortiz |
| Rigueiro Veloso,  Pedro. MD, PhD* | Álvarez Barredo, María.  PhD  Álvarez Dobaño, José  Manuel. MD, PhD  Rábade Castedo, Carlos. PhD | Complejo Hospital Clinico Universitario de Santiago de Compostela, Servicio de Cardiologia, C/ Choupana s/n, Santiago de Compostela, A Coruna, 15706, Spain | Hospital 12 de Octubre, Ctra de Andalucia, Km 5.4 Hospital 12 Octubre, Madrid, 28041, Spain  Chairperson: De Frutos, Javier Ortiz |
| Román Broto,  Antonio. MD | Bravo Masgoret, Carles. MD  López Meseguer, Manuel. MD  Monforte Torres, Victor. MD, PhD | Hospital Vall d'Hebrón, Servei de Pneumologia Planta Baixa, Paseo Vall de Hebrón 119-129, Barcelona, 8035, Spain | Hospital 12 de Octubre, Ctra de Andalucia, Km 5.4 Hospital 12 Octubre, Madrid, 28041, Spain  Chairperson: De Frutos, Javier Ortiz |
| Santos Luna,  Francisco. MD* | Redel Montero, Javier. PhD  Vaquero Barrios, José  Manuel | Hospital Reina Sofía, Avda. Menéndez Pidal s/n, Córdoba, 14004, Spain | Hospital 12 de Octubre, Ctra de Andalucia, Km 5.4 Hospital 12 Octubre, Madrid, 28041, Spain  Chairperson: De Frutos, Javier Ortiz |
| Segovia Cubero,  Javier. MD, PhD | Gómez Bueno, Manuel.  PhD | Hospital Puerta de Hierro, C/ Manuel de Falla, 1, Majadahonda Madrid, 28222, Spain | Hospital 12 de Octubre, Ctra de Andalucia, Km 5.4 Hospital 12 Octubre, Madrid, 28041, Spain  Chairperson: De Frutos, Javier Ortiz |
| Sueiro Bendito,  Antonio. MD, PhD* | Gaudó Navarro, Javier. MD  Kopecna, Dita. MD | Hospital Ramón y Cajal, Respiratory Department, Ctra.de Colmenar Viejo km.9,100, Madrid, 28034, Spain | Hospital 12 de Octubre, Ctra de Andalucia, Km 5.4 Hospital 12 Octubre, Madrid, 28041, Spain  Chairperson: De Frutos, Javier Ortiz |
| Valldeperas Combas,  Joan. MD, PhD | None | Hospital Universitario de Bellvitge, C/ Feixa Larga,s/n, L'Hospitalet de Llobregat, 8907, Spain | Hospital 12 de Octubre, Ctra de Andalucia, Km 5.4 Hospital 12 Octubre, Madrid, 28041, Spain  Chairperson: De Frutos, Javier Ortiz |
| Zurbano Goñi, Felipe.  MD, PhD | Ferrer Pargada, Diego  Iturbe Fernández, David | Hospital Marques de Valdecilla, Avd. de Marques de Valdecilla s/n, Santander, 39008, Spain | Hospital 12 de Octubre, Ctra de Andalucia, Km 5.4 Hospital 12 Octubre, Madrid, 28041, Spain  Chairperson: De Frutos, Javier Ortiz |
| **Sweden** |  |  |  |
| Hubbert, Laila. MD,  PhD  Jansson, Kjell. MD (FPI) | Alfetlawi, Monthir. MD  Enell, Eva-Lena. MD | Universitetssjukhuset i Linköping, Kardiologiska kliniken, Universitetssjukhuset i Linköping, Linköping, SE-581 85, Sweden | Regionala Etikprövningsnämnden I Göteborg, Guldhedsgatan 5A, Göteborg, SE-413 20, Sweden  Chairperson: Kärrström, Margit |
| Rådegran, Göran. MD  Ekmehag, Björn LV. MD (FPI) | Hesselstrand, Roger. MD  Holm, Johan  Kornhall, Björn. MD  Reitan, Öyvind. MD | Skånes universitetssjukhus, Kliniken för hjärtsvikt och klaffsjukdom, Lund, Division  H2LK, Hjärtmottagningen, Skånes universitetssjukhus, Lund, SE-221 85, Sweden | Regionala Etikprövningsnämnden I Göteborg, Guldhedsgatan 5A, Göteborg, SE-413 20, Sweden  Chairperson: Kärrström, Margit |
| Rundqvist, Bengt. MD  Selimovic, Nedim. MD, PhD (FPI) | Andersson, Bert. MD  Bartfay, Sven-Erik Bollano, Entela  DuttaRoy, Smita. MD Gilljam, Thomas. MD Sakiniene, Egidija. MD | Sahlgrenska Universitetssjukhuset, Kardiologens forskningsenhet, Vån 5, Gröna Stråket 9, Göteborg, SE-413 45, Sweden | Regionala Etikprövningsnämnden I Göteborg, Guldhedsgatan 5A, Göteborg, SE-413 20, Sweden  Chairperson: Kärrström, Margit |
| Söderberg, Stefan.  MD, PhD | Gonzalez, Manuel. MD | Umea University Hospital, Norrlands Universitetssjukhus, Medicinkliniken, Department of Public Health and Clinical Medicine, Cardiology, Norrlands Universitetssjukhus, Umeå, SE-901 85, Sweden | Regionala Etikprövningsnämnden I Göteborg, Guldhedsgatan 5A, Göteborg, SE-413 20, Sweden  Chairperson: Kärrström, Margit |
| Wikström, Gerhard G. MD, PhD | Björklund, Erik. MD  Christersson, Christina. MD  Kavianipour, Mohammad. MD | Akademiska Sjukhuset, Kardiologkliniken, Akademiska Sjukhuset, Uppsala, SE-751 85, Sweden | Regionala Etikprövningsnämnden I Göteborg, Guldhedsgatan 5A, Göteborg, SE-413 20, Sweden  Chairperson: Kärrström, Margit |
| **United Kingdom** |  |  |  |
| Coghlan, John  Gerard. MD, FRCP | Dobarro, David  Lynch, Bernadette Schreiber, Benji Schwaiger, Johannes  Valerio, Christopher | Royal Free Hospital, Pulmonary Vascular Disease Unit, Pond Street, London, NW3 2QH, United Kingdom | Western Infirmary, West of Scotland REC1, Ground Floor, Tennent Institute, 38 Church Street, Glasgow, G11 6NT, United Kingdom  Chairperson: Hunter, John |
| Kiely, David. MD,  FRCP | Condliffe, Robin  Elliot, Charles | Royal Hallamshire Hospital, Pulmonary Vascular Disease Unit, Room M39A, Glossop Road, Sheffield, S10 2JF, United Kingdom | Western Infirmary, West of Scotland REC1, Ground Floor, Tennent Institute, 38 Church Street, Glasgow, G11 6NT, United Kingdom  Chairperson: Hunter, John |
| Peacock, Andrew J.  MD, FRCP | Brash, Lauren  Brewis, Melanie  Church, Colin  Crawley, Stephen  Johnson, Martin K  Ling, Yi  McGlinchey, Neil Thomson, Stephen | Golden Jubilee National Hospital, Scottish Pulmonary Vascular Unit, Agamemnon Street, Clydebank, G81 4DY, United Kingdom | Western Infirmary, West of Scotland REC1, Ground Floor, Tennent Institute, 38 Church Street, Glasgow, G11 6NT, United Kingdom  Chairperson: Hunter, John |
| Pepke-Zaba, Joanna.  PhD, FRCP | Hagen, Guy  Hadinnapola, Charaka  Kacprzak, Aneta  Sheares, Karen  Taboarda Buasso, Dolores | Papworth Hospital, Papworth Everard, Cambridge, Cambridgeshire, CB3 8RE, United Kingdom | Western Infirmary, West of Scotland REC1, Ground Floor, Tennent Institute, 38 Church Street, Glasgow, G11 6NT, United Kingdom  Chairperson: Hunter, John |
| Wort, Stephen John.  MBBS, FRCP, PhD* | Dimopoulis, Konstantinos  Marino, Philip | Royal Brompton and Harefield NHS Foundation Trust, Biomedical Research Unit, Sydney Street, London, SW3  6NP, United Kingdom | Western Infirmary, West of Scotland REC1, Ground Floor, Tennent Institute, 38 Church Street, Glasgow, G11 6NT, United Kingdom  Chairperson: Hunter, John |
| **United States** |  |  |  |
| Allen, Roblee. MD | Albertson, Timothy. MD  Avdalovic, Mark. MD Whitcomb, Charles K. MD | University of California Davis Medical Center, 2315 Stockton Boulevard, Room 5212, Sacramento, California, 95817, United States | Institutional Review Board, 2315 Stockton Boulevard, Sacramento, California, 95817, United States  Chairperson: Hahn, Cynthia |
| Allen, Samuel  Abraham. DO | Asadulla, Mohammed S.  MD | William Beaumont Hospital, 44201 Dequindre Road, Troy, Michigan, 48085, United States | William Beaumont Hospital, Human Investigation Committee, Pharmacy Dock #1, 3811 West Thirteen Mile Road, Royal Oak, Michigan, 48073-6769, United States  Chairperson: Koerber, John M. PharmD |
| Aris, Robert M. MD | Fares, Wassim. MD  Ford, Hubert James. MD | University of Colorado Cardiac and Vascular Center, Anschutz Inpatient Pavillion, 12605 East 16th Avenue Room 3.2203, Aurora, Colorado, 80045, United States  University of Colorado Health Sciences Center, 12401 East 17th Avenue, Aurora, Colorado, 80045, United States | University of North Carolina, Office of Human Research Ethics-Biomedical Institutional Review Board, CB# 7097, Medical School Building 52, Mason Farm Road, Chapel Hill, North Carolina, 27599, United States  Chairperson: Humphry, Ruth |
| Badesch, David B.  MD, FACP, FCCP | Abaca, Etta  Abbott, Cheryl. RN  Arnez, Gentle. RN, BSN  Bull, Todd M. MD Dempsey, Edward C. MD  Graham, Brian. MD  Kuiphoff, Brenda. RN, BSN  McCollister, Deborah. RN, BSN  Zupancic, Debra. FNP | University of Colorado Health Sciences Center, 12401 East 17th Avenue, Aurora, Colorado, 80045, United States | Colorado Multiple Institutional Review Board (COMIRB), 13001 East 17th Place, Room No 3214, Aurora, Colorado, 80010, United States  Chairperson: Easterday, Ken |
| Bajwa, Abubakr A.  MD, FCCP | Cury, James. MD  Jones, Lisa M. MD  Loft, Levi C. BS  Shujaat, Adil. MD  Usman, Faisal. MD  Wood, Janice. RRT | University of Florida Health Science Center, College of Medicine, 653 West 8th Street, Faculty Clinic, 3rd Floor, Jacksonville, Florida, 32209, United States | Western Institutional Review Board (WIRB), 1019 39th Avenue South East, Puyallup, Washington, 98374, United States  Chairperson: Wilkins, R Bert |
| Benza, Raymond L.  MD | Agarwal, Richa. MD  Kanwar, Manreet. MD Murali, Srinivas. MD, FACC, FACP  Raina, Amresh. MD  Ravi, Pranav. MBBS, CCRC, CCRP  Rossi, Joan. RN, BSN  Sokos, George G. DO | Allegheny General Hospital, 320 East North Avenue, Pittsburgh, Pennsylvania, 15212, United States | Allegheny Singer Research Institute-West Penn Allegheny Health System Institutional Review Board (ASRI- WPAHS-IRB), 320 East North Avenue, Pittsburgh, Pennsylvania, 1521, United States  Chairperson: Parda, David. MD |
| Bourge, Robert  Charles. MD | Acharya, Deepak. MD  Cadeiras, Martin. MD  Loyaga-Rendon, Renzo Y.  MD  Pamboukian, Salpy V. MD, MSPH  Tallaj, Jose A. MD, FACC | The Kirklin Clinic, 2000 Sixth Avenue South, Birmingham, Alabama, 35233, United States  Zeigler Research Building, 703  South 191th Street, 535 Birmingham, Alabama, 35294, United States | Western Institutional Review Board (WIRB), 1019 39th Avenue South East, Puyallup, Washington, 98374, United States  Chairperson: Wilkins, R Bert |
| Chakinala, Murali  Mohan. MD | Billadello, Joseph J. MD  Cedars, Ari M. MD Duncan, Maribeth. APRN  Newton-Lovato, Ellen. RN, BSN | Washington University School of Medicine, Division of Pulmonary and Critical Care, Campus Box 8052, 660 South Euclid Avenue, Saint Louis, Missouri, 63110, United States | Washington University School of Medicine, Human Research Protection Office, 660 South Euclid Avenue, Campus Box 8089, Saint Louis, Missouri, 63110, United States  Chairperson: FWA00002284 |
| Costanzo, Maria  Rosa. MD | Box, Laura. RN  Digate, Mary. MSN, CCNS  Elder, Lea. RN, MSN, MBA, CCRC  Kern, Sarah  Paprockas, Kim Sabaliauskas, Gaile. MD  Valika, Ali. MD  Yanz, Joan. RN, BSN | Midwest Heart Foundation, 1901 South Meyers Road, Oakbrook Terrace, Illinois, 60181, United States | Copernicus Group Institutional Review Board (CGIRB), One Triangle Drive, Suite 100, P.O. Box 110605, Research Triangle Park, North Carolina, 27709  Chairperson: Veit, Glenn C. JD |
| Criner, Gerard J. MD* | Allen, Denean. BS  Desai, Parag. MD Johnson, Sylvia. RN Mamary, Albert James. MD  Satti, Aditi. MD  Shenoy, Kartik. MD  Weaver, Sheila. DO | Temple Lung Center, Parkinson Pavilion, Philadelphia, Pennsylvania, 19140, United States | Western Institutional Review Board (WIRB), 1019 39th Avenue South East, Puyallup, Washington, 98374, United States  Chairperson: Wilkins, R Bert |
| Cummings, Rhett J.  MD | Bowerfind, William. MD  Chesnutt, Asha. MD  Day, Mary Alice (Meg) L. CCRP  Heffner, John. MD  Hotchkin, David L. MD  Jacobs, Marc A. MD  Lefor, Michael J. MD  Libby, Louis S. MD  Loos, Arlena. CRC  Morganroth, Melvin. MD  Schaumberg, Thomas H. MD  Skokan, Michael D. MD Strauss, Wayne. MD Wesenberg, Karen. MD | The Oregon Clinic, PC/ Pulmonary Division, 1111 North East 99th Avenue, Suite #200, Portland, Oregon, 97220, United States | Copernicus Group Institutional Review Board (CGIRB), One Triangle Drive, Suite 100, P.O. Box 110605, Research Triangle Park, North Carolina, 27709  Chairperson: Veit, Glenn C. JD |
| DeBoisblanc, Bennett Paul. MD | Ali, Murtuza. MD  Lammi, Matthew. MD  Jain, Surma. MD Tejedor, Richard. MD  Ventura, Hector. MD | Louisiana State University Health Sciences Center-New Orleans, Department of Internal Medicine, Section of Pulmonary/Critical Care Medicine, 1901 Perdido Street, Suite 3205, New Orleans, Louisiana, 70112, United States | Louisiana State University Health Sciences Center-New Orleans, Institutional Review Board, 433 Bolivar Street, New Orleans, Louisiana, 70112, United States  Chairperson: Kartz, Ken. MD |
| Eggert, Michael S.  MD, FCCP* | Eich, David M. MD  Garnett, Alfred. MD Mahoney, Paul D. MD Mckechnie, Ronald S. MD  Muntzer, Laura. PA  Rozek, Robyn. PA Tomlinson, James. MD | Sentara Cardiovascular Research Institute, Division of Pulmonary and Critical Care Medicine, Sentara Heart Hospital, 600 Gresham Drive, Suite 8630, Norfolk, Virginia, 23507, United States | Copernicus Group Institutional Review Board (CGIRB), One Triangle Drive, Suite 100, P.O. Box 110605, Research Triangle Park, North Carolina, 27709  Chairperson: Veit, Glenn C. JD |
| Elliott, Charles  Gregory. MD | Boekweg, Deedre Elizabeth. RN, BSN  Brown, Lynette Mardel. MD, PhD  Hayes, Michael Lee. RN, BSN  Hegewald, Matthew James. MD  Kitterman, Natalie Jo Seegmiler. RN  Tomer, David Peter. MS | Intermountain Medical Center, Heart & Lung Centre, 5121 South Cottonwood Street, P.O. Box 577000, Murray, Utah, 84157-7000, United States | Intermountain Healthcare Urban Central Region Institutional Review Board, LDS Hospital, Medical Staff Office, 8^th^ Avenue & C Street, SaIt Lake City, Utah, 84143, United States  Chairperson: Musci, Anthony G |
| Elwing, Jean Marie.  MD, FCCP | Lanich, Amber. ACNP-BC  Panos, Ralph. MD | University Hospital, 234 Goodman Avenue, Cincinnati, Ohio, 45219, United States  University of Cincinnati Physician Medical Arts Building, 222 Piedmont Avenue, Suite 4000, Cincinnati, Ohio, 45219, United States | Western Institutional Review Board (WIRB), 1019 39th Avenue South East, Puyallup, Washington, 98374, United States  Chairperson: Wilkins, R Bert |
| Engel, Peter J. MD,  FACC | Bailey, Kimberly A. MD  Bruce, Jeremy E. MD  Kereiakes, Dean J. MD  Wahl, Dain. DO | The Carl and Edyth Lindner Center for Research and Education at the Christ Hospital, Suite 424, 2123 Auburn Avenue, Cincinnati, Ohio, 45219, United States | Copernicus Group Institutional Review Board (CGIRB), Suite 100, One Triangle Drive, P.O. Box 110605, Research Triangle Park, North Carolina, 27709, United States  Chairperson: Veit, Glenn C. JD |
| Fagan, Karen A. MD | Schaphorst, Kane L. MD | University of Southern Alabama Medical Centre, 2451 Fillingim Street, Suite 10-G, Mobile, Alabama, 36617, United States | University Of South Alabama Institutional Review Board, College of Medicine, Institutional Review Board Office, Hsb Suite 1600, 307 N University Boulevard, CSAB # 138, Mobile, Alabama, 36688, United States  Chairperson: Russell, John. MD |
| Farber, Harrison W.  MD | Klings, Elizabeth. MD | Boston Medical Center/Boston University School of Medicine Center Pulmonary, Doctors Office Building, 720 Harrison Avenue, 4th Floor, Boston, Massachusetts, 02118, United States | Western Institutional Review Board, 1019 39th Avenue South East, Puyallup, Washington, 98374, United States  Chairperson: Wilkins, R Bert. MD |
| Farmer, Mary Jo S.  MD, PhD | Tidswell, Mark. MD | Baystate Pulmonary & Critical Care Medicine, 3300 Main Street, Springfield, Massachusetts, 01199, United States | Current: Baystate Medical Center Institutional Review Board, 759 Chestnut Street, Springfield, Massachusetts, 01199, United States  Chairperson: Richardson, Matthew  Initial: Saint Vincent Hospital/ Reliant Medical Group/Fallon Community Health Plan Institutional Review Board, 123 Summer Street, Worcester, Massachusetts, 01608, United States  Chairperson: Black, Robert |
| Feldman, Jeremy P.  MD | Ahearn, Greogory S. MD | Arizona Pulmonary Specialists,  Limited, Suite 300, 3330 North 2^nd^ Street, Phoenix, Arizona, 85012, United States | Copernicus Group Institutional Review Board (CGIRB), Suite 100, One Triangle Drive, P.O. Box 110605, Research Triangle Park, North Carolina, 27709, United States  Chairperson: Veit, Glenn C. JD |
| Fisher, Micah R. MD | Gillespie, Jane. RN  Jacob, Christine. APRN  Quintero, David. MD Zemeer, Patrick. PA | The Emory Clinic, 1365 Clifton Road, Building A, Atlanta, Georgia, 30322, United States | Western Institutional Review Board, 1019 39th Avenue South East, Puyallup, Washington, 98374, United States  Chairperson: Wilkins, R Bert. MD |
| Gerke, Alicia K. MD, MS  Hansdottir, Sif. MD, PhD (FPI) | Cadaret, Linda. MD  Campbell, Jennifer Goerbig. MD  Cotarlan, Vlad. MD Frances L, Johnson. MD Groskreutz, Dayna. MD Khurram, Irfan. MD McGroary, KellyAnn Light. MD  Schadler, Angela. ARNP, RN  Smith, Elisa. ARNP | University of Iowa Hospitals and Clinics, 200 Hawkins Drive, Iowa City, Iowa, 52242, United States | Western Institutional Review Board, 1019 39th Avenue South East, Puyallup, Washington, 98374, United States  Chairperson: Wilkins, R Bert. MD |
| Grinnan, Daniel C.  MD | Fairman, R Paul. MD  Frayser, Amy  Gotico Reyes, Yvonne Pinson, Janet. RN  Usry, Lou | Virginia Commonwealth University Medical Center, 1250 East Marshall Street, Richmond, Virginia, 23298, United States | Western Institutional Review Board, 1019 39th Avenue South East, Puyallup, Washington, 98374, United States  Chairperson: Wilkins, R Bert. MD |
| Harvey, William L F.  MD | Becka, Susan W. RN, BSN, CCRN, CCRC  Broach, Debra L, RN, BSN  Ford, Anne L. MD  Isaacs, Tonya G. RN, BSN, CCRC  Jackson, Judith A. RN, BSN, CCRC  Logan, Betty. RN, BSN,  CCRC  McNamee, Ronda. RN, BSN, CCRC  Miracle, Nancy E. RN, BSN, CCRC  Oakes, Tessa R. RN Strickland, Terri L. RN, BSN, CCRC  Thorp, Anne-Marie. RN, BSN, CCRC  Turk, Erin, RN, BSN | Clarian Health, 11725 Illinois Street, Suite 465, Carmel, Indiana, 46032, United States | Indiana University Institutional Review Board, Indiana University Human Subjects Office, Indianapolis, Suite 618, 620 Union Drive, Indianapolis, Indiana, 46202, United States  Chairperson: Schilder, Jeanne M. MD |
| Hassan, Alnuaimat M. MD, MBBS | Carrie, Robin D. ARNP  Faruqi, Ibrahim. MD  Jantz, Michael A. MD | Shands at the University of Florida, 1600 South West Archer Road, Box 100225, Gainesville, Florida, 32610, United States | Western Institutional Review Board, 1019 39th Avenue South East, Puyallup, Washington, 98374, United States  Chairperson: Wilkins, R Bert. MD |
| Hill, Nicholas S. MD | Garza, Ana. MD  Jothianandan, Karthik. MD  Preston, Ioana. MD Roberts, Kari. MD  Visnaw, Karen. RN | Tufts Medical Center, 800 Washington Street, Boston, Massachusetts, 02111, United States | Tufts Medical Center Institutional Review Board, Hematology/Oncology #245, 800 Washington Street, Box 817, Boston, Massachusetts, 02111, United States  Chairperson: Klein, Andreas K. MD |
| Horn, Evelyn M. MD | Avrahami, Neshama. RN,  NP-c  Gadioma, Rosemarie. NP  Garcia, Nicole. MS, ANP- BC, CCRN  Gjerde, Cecilie. RN, FNP- BC  Jorgensen, Birgit. NP  Sobol, Irina. MD Yushak, Madeline. RN | Weill Cornell Medical Center, Perkin Center for Heart Failure, Suite 443-Starr 4, 520 East 70th Street, New York, New York, 10021, United States | Weill Cornell Medical College Institutional Review Board, 407 East 61st Street, RR-110, New York, New York, 10065, United States  Chairperson: Behrman, David  Chairperson: Goldstein, Peter. MD |
| Kim, Hyong S. MD | Papamatheakis,  Demosthenes. MD | University of California, San Diego, 2nd Floor, 2-042, 9444 Medical Center Drive, La Jolla, California, 92093, United States | University of California, San Diego, Human Research Protections Program, East Campus Office Building, First Floor, 9444 Medical Center Drive, La Jolla, California, 92093, United States  Chairperson: Magit, Anthony. MD |
| Klinger, James R. MD | Baillargeon, Erica. NP  Houtchens, Jeanne E, NP-C  Martin, Douglas W. MD Ventetuolo, Corey E. MD | Rhode Island Hospital, 593 Eddy Street, Providence, Rhode Island, 02903, United States | Rhode Island Hospital Committee for the Protection of Human Subjects, 167 Point Street, Coro West Suite 1.300, Providence, Rhode Island, 02903, United States  Chairperson: Linakis, James. MD |
| Mathier, Michael A.  MD | Alvarez, Rene. MD  Cadaret, Linda. MD Champion, Hunter. MD | University of Pittsburgh Medical center, 200 Lothrop Street, Pittsburgh, Pennsylvania, 15213-2582, United States | Western Institutional Review Board, 1019 39th Avenue South East, Puyallup, Washington, 98374, United States  Chairperson: Wilkins, R Bert. MD |
| Michaelson, Jeffrey E. MD, FCCP, FAASM* | Maslanka, Nicole. RN, BSN  Rosenthal, Steven. MD  Hernon, Michaelle C. CRNP  Ishizawar, David. MD Keeley, Jennifer H. CRNP  Kliner, Jennifer. CRNP McGrath, Paula. RN McNamara, Dennis. MD  Pollera, Mary. RN  Ramani, Ravi. MD  Simon, Marc. MD Teuteberg, Jeffrey. MD Zhang, Zhong (Joan). MD, CRNP | Atlanta Institute for Medical Research, Inc., Suite 220, 495 Winn Way, Decatur, Georgia, 30030, United States | Copernicus Group Institutional Review Board (CGIRB), Suite 100, One Triangle Drive, P.O. Box 110605, Research Triangle Park, North Carolina, 27709, United States  Chairperson: Veit, Glenn C. JD |
| Migliore, Christina.  MD, FCCP  Studer, Sean M. MD, FCCP (FPI) | Baran, David A. MD, FACC  Williams, Juliet. CCRC | Barnabas Health Newark Beth Israel Medical Center, 201 Lyons Avenue, Suite C-1, Newark, New Jersey, 07112, United States | Barnabas Health Newark Beth Israel Medical Center Institutional Review Board, 201 Lyons Avenue, H-3, Newark, New Jersey, 07112, United States  Chairperson: Parsonnet, Victor. MD |
| Minkin, Ruth. MD | Eden, Edward. MD  Tartell, Lori. RN | Saint Luke's - Roosevelt Hospital Center, 1000 10th Avenue – Room 3A-55, New York, New York, 10019, United States | Saint. Luke's Roosevelt Hospital Center, Institute for Health Sciences, Institutional Review Board, 432 West 58th Street - Room 207, New York, New York, 10019, United States  Chairperson: Bania, Theodore. MD |
| Mobin, Syed I. MD,  FCCP, DABSM, FAASM | Garcia, Ruel B. MD, FCCP  Haim, Y Daniel. MD, FCCP  Layish, Daniel T. MD, FACP, FCCP, FAASM  Masood, Ahmed. MD, FCCP | Central Florida Pulmonary Group P.A, 1115 East Ridgewood Street, Orlando, Florida, 32803, United States | Copernicus Group Institutional Review Board (CGIRB), Suite 100, One Triangle Drive, P.O. Box 110605, Research Triangle Park, North Carolina, 27709, United States  Chairperson: Veit, Glenn C. JD |
| Oudiz, Ronald J. MD | Budoff, Matthew J. MD | Los Angeles Biomedical Research Institute at Harbor UCLA Medical Centre, 1124 West Carson Street, E6/E5, Torrance, California,  90502, United States | Western Institutional Review Board (WIRB), 1019 39th Avenue South East, Puyallup, Washington, 98374, United States  Chairperson: Wilkins, R Bert |
| Parambil, Joseph. MD  Minai, Omar A. MD (FPI) | Becerra, Mario. BA  Heresi, Gustavo. MD  Timmerman, Kasi Zak, Katie. BS | Cleveland Clinic Foundation, 9500 Euclid Avenue A-90, Cleveland, Ohio, 44195, United States | Cleveland Clinic Institutional Review Board OS-1, Cleveland Clinic, 9500 Euclid Avenue, Cleveland, Ohio, 44195, United States  Chairperson: Lichtin, Alan |
| Park, Myung H. MD | Ramani, Guatam V. MD | University of Maryland Medical Centre, 22 South Greene Street, Baltimore, Maryland, 21201, United States | University of Maryland School of Medicine, Human Research Protections Office (HRPO), UMB BioPark, Building One, 800 West Baltimore Street, Suite 100, Baltimore, Maryland, 21201, United States  Chairperson: Rosenthal, Robert |
| Rahaghi, F Franck.  MD | Ferrer, Gustavo. MD  Mehta, Jinesh. MD  Navas, Elsy V. MD Okafor, Ndubuisi. MD | Cleveland Clinic Florida - Weston, 2950 Cleveland Clinic Boulevard, Weston, Florida, 33331, United States | Institutional Review Board, Cleveland Clinic Foundation, 9500 Euclid Avenue, Cleveland, Ohio, 44195, United States  Chairperson: Beyer, Daniel |
| Rischard, Franz. DO | Knoper, Steven R. MD | University of Arizona Clinical and Translational Science (CATS) Research Center, 1515 North Campbell Avenue, Room 1925U, Tucson, Arizona, 85724, United States | Western Institutional Review Board (WIRB), 1019 39th Avenue South East, Puyallup, Washington, 98374, United States  Chairperson: Wilkins, R Bert |
| Robbins, Ivan M. MD | Gonzalez, Shelly. LPN  Hemnes, Anna. MD  Lechman, Andja. RN  Oyler, Tracy L. RN  Pugh, Meredith. MD | Vanderbilt University Medical Center, 1161 21st Avenue South, T1218 MCN, Nashville, Tennessee, 37232, United States | Vanderbilt University, Institutional Review Board, 504 Oxford House, Nashville, Tennessee, 37232, United States  Chairperson: Girard, Timothy |
| Rosenthal, Steven M.  MD, FACC* | Maslanka, Nicole. RN  Michaelson, Jeffrey E. MD | Atlanta Institute for Medical Research Inc., Suite 220, 495 Winn Way, Decatur, Georgia, 30030, United States | Copernicus Group Institutional Review Board, Suite 100, One Triangle Drive, P. O. Box 110605, Research Triangle Park, North Carolina, 27709, United States  Chairperson: Veit, Glenn |
| Rosenzweig, Erika B. MD | Brady, Daniela. MD  Kerstein, Diane. MD Krishnan, Usha. MD  Mituniewicz, Johnell. MD  Whee, Katherine. RN  Zuckerman, Warren. MD | Columbia University Medical Center, 3959 Broadway, BH2N, New York, New York, 10032, United States | Columbia University Medical Center, Institutional Review Board, 722 West  168th Street, 4th Floor, New York, New York, 10032, United States  Chairperson: Rutuolo, Brenda |
| Rubenfire, Melvyn.  MD* | Burks, Marsha. RN  Davis, Melinda. MD McLaughlin, Vallerie. MD  Robb, Heidi. RN  Visovatti, Scott. MD | University of Michigan Medical Center, 1500 East Medical Center Drive, CVC 2nd Floor 2386 A, Ann Arbor, Michigan, 48109, United States | IRBMED, 2800 Plymouth Road, Building 200, Room 2086, Ann Arbor, Michigan, 48109-28003, United States  Chairperson: Sugar, Alan. MD |
| Safdar, Zeenat. MD,  FACP | Frost, Adaani. MD | Baylor College of Medicine, 6620 Main Street, Houston, Texas, 77030, United States | Baylor College of Medicine, Office of Research, Room 600 D, One Baylor Plaza, Houston, Texas, 77030, United States  Chairperson: Habib, Gabriel. MD |
| Satterwhite, Lewis G. MD  Williamson, Timothy L. MD, FCCP (FPI) | Couldry, Barbara  Curtis, Leslie  Kaus, Courtney  Nekkalapudi, Srilakshmi  Olson, Tammy  Schooley, Adam  Spikes, Leslie. MD  Stites, Steven W. MD  Thomas-Dodson, Stephanie  Watson, Marla E | University of Kansas Medical Center, 3901 Rainbow Boulevard, Kansas City, Kansas, 66160-7820, United States | Human Subjects Committee, University of Kansas Medical Center, 3901 Rainbow Boulevard, Kansas City, Kansas, 66160, United States  Chairperson: Schroeder, Valerie. MD |
| Saydain, Ghulam. MD | Dogra, Sanjay. MD  Krell, Willane. MD | Wayne State University, Detroit Medical Centre, Suite 3915, 3- Hudson, 3990 John R Street, Detroit, Michigan, 48201, United States | Human Investigation Committee (HIC) Office, 87 East Canfield, 2nd Floor, Detroit, Michigan, 48201, United States  Chairperson: FWA 00002460 |
| Shapiro, Shelley. MD, PhD | Bersohn, Maicolm. MD, PhD  Hill, Wendy. MSN | West Los Angles VA Healthcare Center, Pulmonary Hypertension Program, Cardiology Section 111- E, 11301 Wilshire Boulevard, Building 500, Los Angeles, California, 90073, United States | VA Greater Los Angeles Healthcare System (151- Institutional Review Board), 11301 Wilshire Boulevard, Building 114, Los Angeles, California, 90073, United States  Chairperson: Wallbom, Agnes S. MD |
| Smart, Frank W. MD* | Fearon-Clarke, Jacqueline.  RN  Kleet, Audrey. RN Langlois, Elizabeth. RN | Morristown Memorial Hospital, Gagnon Cardiovascular Institute, 100 Madison Avenue, Morristown, New Jersey, 7962, United States | Atlantic Health Institutional Review Board, 475 South Street, Morristown, New Jersey, 7960, United States  Chairperson: Dise, Craig |
| Smith, Kerri A. MD  Forfia, Paul R. MD (FPI) | Craig, Kimberly  Pickering, Faith  Rame, J Eduardo | Hospital of the University of Pennsylvania, Cardiovascular Research, Penn Tower 6th Floor, 3400 Spruce Street, Philadelphia, Pennsylvania, 19104, United States | University of Pennsylvania Office of Regulatory Affairs, 3624 Market Street, Suite 301 S., Philadelphia, Pennsylvania, 19104, United States  Chairperson: Meagher, Emma |
| Sood, Namita. MD,  FCCP | Bhatt, Nitin. MD  Daniels, Curtis J. MD  Ezzie, Michael E. MD  Franco, Veronica. MD | OSU Martha Morehouse Medical Plaza, 2050 Kenny Road, Suite 2600, Columbus, Ohio, 43221, United States | Western Institutional Review Board (WIRB), 1019 39th Avenue South East, Puyallup, Washington, 98374, United States  Chairperson: Wilkins, R Bert |
| Spradley, Christopher D. MD, FCCP | Hyatt, Eve  Sanchez, Juan. MD | Scott & White Memorial Hospital and Clinic, 2401 South 31st Street, Temple, Texas, 76508, United States | Scott & White Institutional Review Board, 2401 South 31st Street, Temple, Texas, 76508, United States  Chairperson: FWA00003358 |
| Sulica, Roxana. MD* | Fenton, Rebecca. RN  Kuntsevich, Viktoryia. PhD | Beth Israel Medical Center, 10 Union Square East, Suite 2A, New York, New York, 10003, United States | Beth Israel Medical Center Institutional Review Board, 24th Floor, 160 Water Street, New York, New York, 10038, United States  Chairperson: Winston, Arnold |
| Talwar, Arunabh. MD, FCCP | Dedopoulos, Sophy. MD,  NP  Sadoughi, Ali. MD | North Shore - LIJ Health System, Suite 107, 410 Lakeville Road, New Hyde Park, New York, 11040, United States | Biomedical Research Alliance of New York LLC, Institutional Review Board, 1981 Marcus Avenue, Suite 210, Lake Success, New York, 11042, United States  Chairperson: Hahn, Cynthia |
| Torres, Fernando. MD | Chin, Kelly. MD  Harden, Scarlett. ACNP  Kingman, Martha. FNPC  Patrizi, Robert. MD | University of Texas Southwestern Medical Center at Dallas, Cardiovascular Institute/Pulmonary Hypertension Clinic, 5939 Harry Hines Boulevard, POB II, Suite 600, Dallas, Texas, 75390, United States | University of Texas Southwestern Medical Center Institutional Review Board, 5323 Harry Hines Boulevard, Dallas Texas, 75390-8843, United States  Chairperson: FWA 00005087 |
| Trichon, Benjamin H. MD, FACC | Abernethy III, William B. MD  Blakely, Jan C. MD Brockwell, Heather M. RN Brown, Linda S. MD Cauthren, Tia G. RN Gibson, Amy E. RN  Hull, Amber B. RN  Lim, Olivia J. RN Murdock, Karen. RN Onder, Rebecca L. RN Pfeiffer, Elisabeth W. RN  Putnam, Natasha C. RN Unks, D (Dennis) Michael. MD  Vaughn, Jackie W. RN Westcott, Susan K. RN | Asheville Cardiology Associates, 5 Vanderbilt Park Dr., Asheville, North Carolina, 28803, United States | Mission Health Institutional Review Board, 509 Biltmore Avenue, Asheville, North Carolina, 28801, United States  Chairperson: Ohmen, Elizabeth |
| Urdaneta-Jaimes,  Jose Antonio. MD, FCCP, FAASM | Daniels, Heather E. ARNP  Lucio, James A MD FCCP  Metcalf, Susan A. ARNPC  Theriault, Vivian M. ARNPBC, MS, CCRN | Pulmonary Disease Specialist Research, Suite A, 1121 North Central Avenue, Kissimmee, Florida, 34741, United States | Copernicus Group Institutional Review Board, Suite 100, One Triangle Drive, Research Triangle Park, North Carolina, 27709, United States  Chairperson: Veit, Glenn |
| Waxman, Aaron B.  MD, PhD | Cockrill, Barbara A. MD  Lawler, Laurie. RN  Schiro, Arlene. NP | Brigham and Women's Hospital, Pulmonary Critical Care Medicine, 75 Francis Street, Harvard Medical School, CPB-3, Boston, Massachusetts, 2115, United States | Partners Human Research Committee, 116 Huntington Avenue, Suite 1002, Boston, Massachusetts, 2115, United States  Chairperson: FWA00000484 |
| Weder, Max M. MD* | Gay, Elizibeth. MD  Robbins, Mark. MD Verghese, George. MD | University of Virginia Medical Center, Division of Pulmonary and Critical Care, Hospital Drive, Private Clinics Building, Charlottesville, Virginia, 22908, United States | University of Virginia Institutional Review Board for Health Sciences Research (IRB-HSR), Suite 400, Box 5, One Morton Drive, Charlottesville, Virginia, 22903, United States  Chairperson: Stevenson, Richard |
| White, James R. MD,  PhD | Frutiger, Karen. RN  Theuer, Alison. RN Weaver, Christine. CCRP  Wessman, Kathleen. RN | University of Rochester Medical Center, Mary Parkes Centre, 400 Red Creek Drive, Suite 110, Rochester, New York, 14623, United States | Western Institutional Review Board, South Hill Business and Technology Centre, 1019 39th Avenue SouthEast, Puyallup, Washington, 98374, United States  Chairperson: Wilkins, R Bert |
| Wichman, Tammy O. MD  Schuller, Dan. MD (FPI) | Brimeyer, Ryan. DO  Gogineni, Vijaya. MBBS  Gupta Pooja. MBBS Keyes, Amy. RRT  Moore, Douglas. MD Morrow, Lee. MD  Pote, Mary. RRT  Ramelb, Theresa. RRT | Creighton University Medical Centre, Division of Pulmonary and Critical Care and Sleep Medicine, 601 North 30th Street, Suite 3820 Omaha, Nebraska, 68131, United States | Creighton University Institutional Review Board, 2500 California Plaza, Omaha, North East, 68178, United States  Chairperson: Kunes-Connell, Mar. PhD |
| Wirth, Joel A. MD,  CM, FCCP | Knauft, Elizabeth M. MD | Maine Medical Center, 22 Bramhall Street, Division of Pulmonary and Critical Care Medicine, Portland, Maine, 04102, United States | Maine Medical Center Institutional Review Board, 81 Research Drive, Scarborough, Maine, 04074, United States  Chairperson: Blaisdell, Laura. MD, MPH |
| Zwicke, Dianne Lynn. MD, FACC | Hastings, Thomas E. DO  Paulus, Sara. PAC Schauer, Paulette. RN Tumuluri, Ramagopal J. MD  Wenzel, Mary. APNP | St Luke's Medical Center, 2900 West Oklahoma Avenue, Milwaukee, Wisconsin, 53215, United States | Aurora Institutional Review Board/ RSPP, 945 North 12th Street, PO Box 342 W310, Milwaukee, Wisconsin, 53201-0342, United States  Chairperson: Anderson, Andy. MD |

*No subjects randomized

All centers participated in the study under the US IND.

**Drug Titration**

Tadalafil was initiated at 20 mg and ambrisentan at 5 mg (whether monotherapy or combination therapy), and drugs were blindly uptitrated at week 4 (tadalafil to 40 mg) and week 8 (ambrisentan to 10 mg).

**Statistical Methods**

We analyzed walk distance using the stratified Wilcoxon rank sum test; missing values were imputed using last observation carried forward unless participants had a death or adjudicated hospitalization for worsening PAH in which case worst rank was imputed. The percentage of patients with a satisfactory clinical response was analyzed as a binary endpoint using logistic regression with no imputation for missing values. Post-hoc analyses of N-terminal pro-B-type natriuretic peptide (NT-proBNP) are presented as the geometric mean and geometric mean ratio and analyzed using mixed models repeated measures with no imputation for missing data.

To address the possibility that the 11 class II participants assigned to monotherapy and ultimately hospitalized were substantially different than the overall group of class II participants, we rank-ordered the 155 Class II participants for both NT-pro-BNP and 6MW. We used descending order for NT-proBNP (low rank means a high value of NT-proBNP); similarly, a low rank for 6MWD means a low value for 6MWD. We assigned ties an average rank. For example, the first tie for 6MWD were the two values between Rank 47 and Rank 50 (i.e., ranks 48 and 49), so we assigned the 2 participants ranks of 48.5. The average (overall) rank was an unweighted average of the two separate ranks.

**Baseline characteristics of eleven Class II participants assigned to monotherapy with a subsequent PAH-related hospitalization**

These participants had baseline characteristics similar to the overall class II group in terms of age, sex, geography, and disease etiology. The average NT-pro-BNP for this group was higher than the overall group and the 6MWD was lower (Supplementary Table 1). To gain further insight into the degree of baseline risk, we ranked the 155 participants separately by NT-pro-BNP (in descending order) and 6MW distance so that the lowest numbers reflected the worst state (highest NT-pro-BNP = lowest rank, lowest walk = lowest rank.) The raw data and ranks of these eleven participants with class II symptoms are shown in Supplementary Table 2. The table illustrates that these 11 subjects have ranks ranging from 1 to 143 (out of 155) for 6MWD and 14 to 117 (out of 144 due to missing values) for NT-proBNP. The average ranks for the 11 participants range from 17 to 106. These 11 participants did have 2 of the worst walks (the worst walk and the 5^th^ worst walk), but there were also relatively higher walks in this group. We conclude that the overall mix was similar to the entire group of Class II participants including all of those assigned to combination therapy (who never had a hospitalization).

**Supplementary Table 1: Baseline Characteristics for the Eleven Class II Participants with a PAH-related Hospitalization**

|  | Class II participants assigned to monotherapy with a PAH-related hospitalization, n = 11; mean ± SD |
| --- | --- |
| Age, years | 52 ± 14 |
| Female | 8 (73%) |
| North America | 5 (46%) |
| Type of PAH |  |
| Idiopathic/ Heritable PAH | 7 (64%) |
| PAH associated with connective tissue disease | 4 (36%) |
| Baseline walk*, m | 360 ± 120 |
| Baseline NT-proBNP*, ng/L | 1200 ± 890 |

*Values rounded to two significant digits for ease of comparison

**Supplementary Table 2: Ranking of Eleven Class II Participants with a PAH-related Hospitalization (baseline values)**

| Participant ID | 6MWD, m | 6MWD Rank | NT-pro-BNP, ng/ml | Pro-BNP Rank | **Overall Rank** |
| --- | --- | --- | --- | --- | --- |
| 281 | 185.9 | 5 | 1611 | 29 | **17** |
| 1302 | 339.5 | 28 | 2614 | 14 | **21** |
| 287 | 130.1 | 1 | 1268 | 42 | **21.5** |
| 203 | 341.7 | 29 | 1387 | 34 | **31.5** |
| 1295 | 396.5 | 70 | 1839 | 22 | **46** |
| 164 | 449 | 116 | 2554 | 15 | **65.5** |
| 130 | 357.2 | 42 | 159 | 117 | **79.5** |
| 894 | 430.5 | 95.5 | 288 | 95 | **95.25** |
| 1086 | 480.5 | 141 | 747 | 61 | **101** |
| 1005 | 424 | 89.5 | 170 | 115 | **102.25** |
| 1035 | 484.5 | 143 | 607 | 69 | **106** |
